# Supplementary figures and images for: Incorporating human mobility to enhance epidemic response and estimate real-time reproduction numbers
Source: PLoS Comput Biol. 2025 Nov 13;21(11):e1013642. doi: 10.1371/journal.pcbi.1013642 (PMC12614531; doi:10.1371/journal.pcbi.1013642)

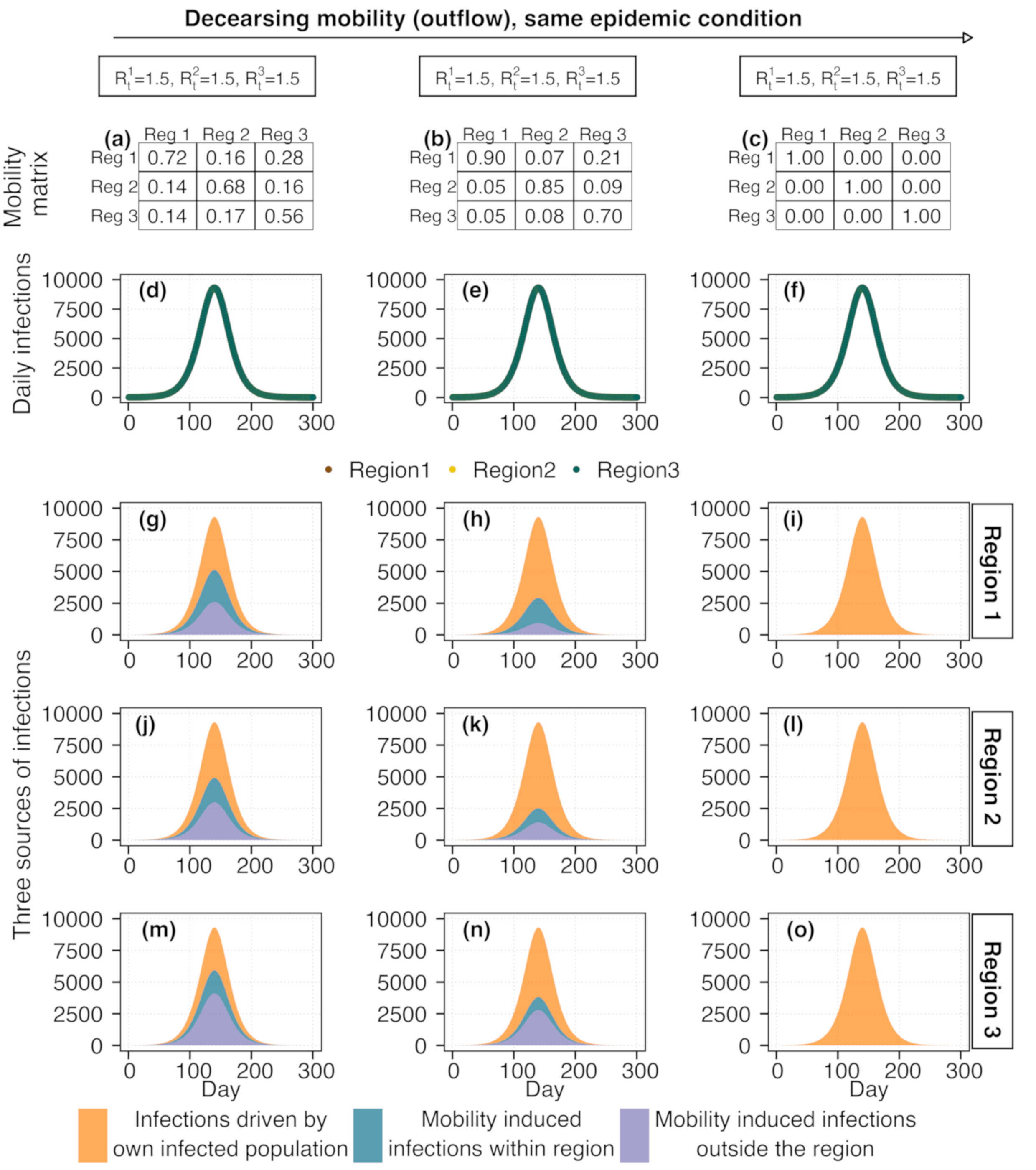

Supplement: S1 Fig — Each region has an equal population of 1,000,000, the mobility matrices and Rt values are specified in the figure. From left to right, we consider the scenarios with decreasing outgoing mobility and increasing non-commuting population. In the rightmost panel of S1 Fig the regions are disconnected (no inter-regional travel), so the mobility matrix is an identity matrix (all off-diagonal elements are zero). S1d-f Fig shows daily infections by region. Since the epidemic conditions are homogeneous, the incidence trajectories are identical across regions even when outflows differ. However, mobility redistributes the location of infection acquisition. S1g-o Fig shows that, although daily case counts are equal for the three regions, infections occur in different destination regions depending on the mobility pattern. (TIFF) [file pcbi.1013642.s001.tif]

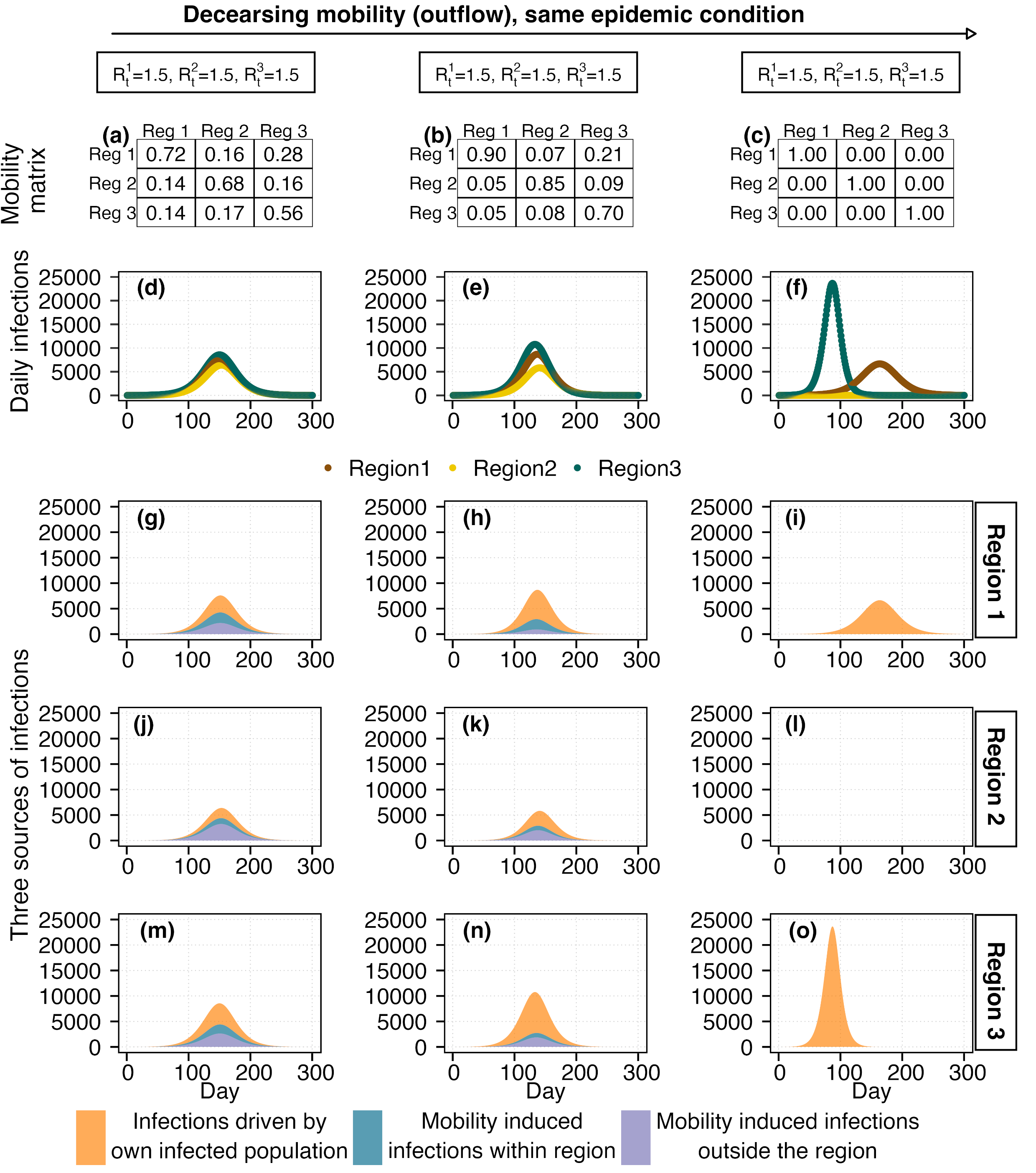

Supplement: S2 Fig — Each region has an equal population of 1,000,000, the mobility matrices and Rt values are specified in the figure. From left to right, we consider the scenarios with decreasing outgoing mobility and increasing non-commuting population. In the rightmost panel of S2 Fig the regions are disconnected (no inter-regional travel), so the mobility matrix is an identity matrix (all off-diagonal elements are zero). S2d–f Fig shows daily infections by region. As the epidemic conditions are heterogeneous over regions, the incidence trajectories are different based on the mobility and local epidemic condition. For example, region 2 is with a controlled situation with Rt<1, however, in S2d–e Fig, there is an outbreak because of the mobility-driven importation, and in the absence of any mobile population it fails to generate any outbreak (See S2f Fig). S2g–o Fig shows the different sources of infections occur in different destination regions depending on the mobility pattern. These simulations underscore that both human mobility and regional epidemic status jointly shape disease transmission dynamics. Mobility has a significant impact in heterogeneous epidemic scenarios, where differences in transmission intensity across regions can lead to outbreaks in low-risk areas through population movement. This is particularly relevant in real-world settings, where such heterogeneity is common during the early stages of an epidemic. As the epidemic becomes widespread and more uniform across regions, local disparities can appear due to the differences in policy implementation, healthcare capacity, demographic characteristics, or socio-economic conditions. (TIFF) [file pcbi.1013642.s002.tif]

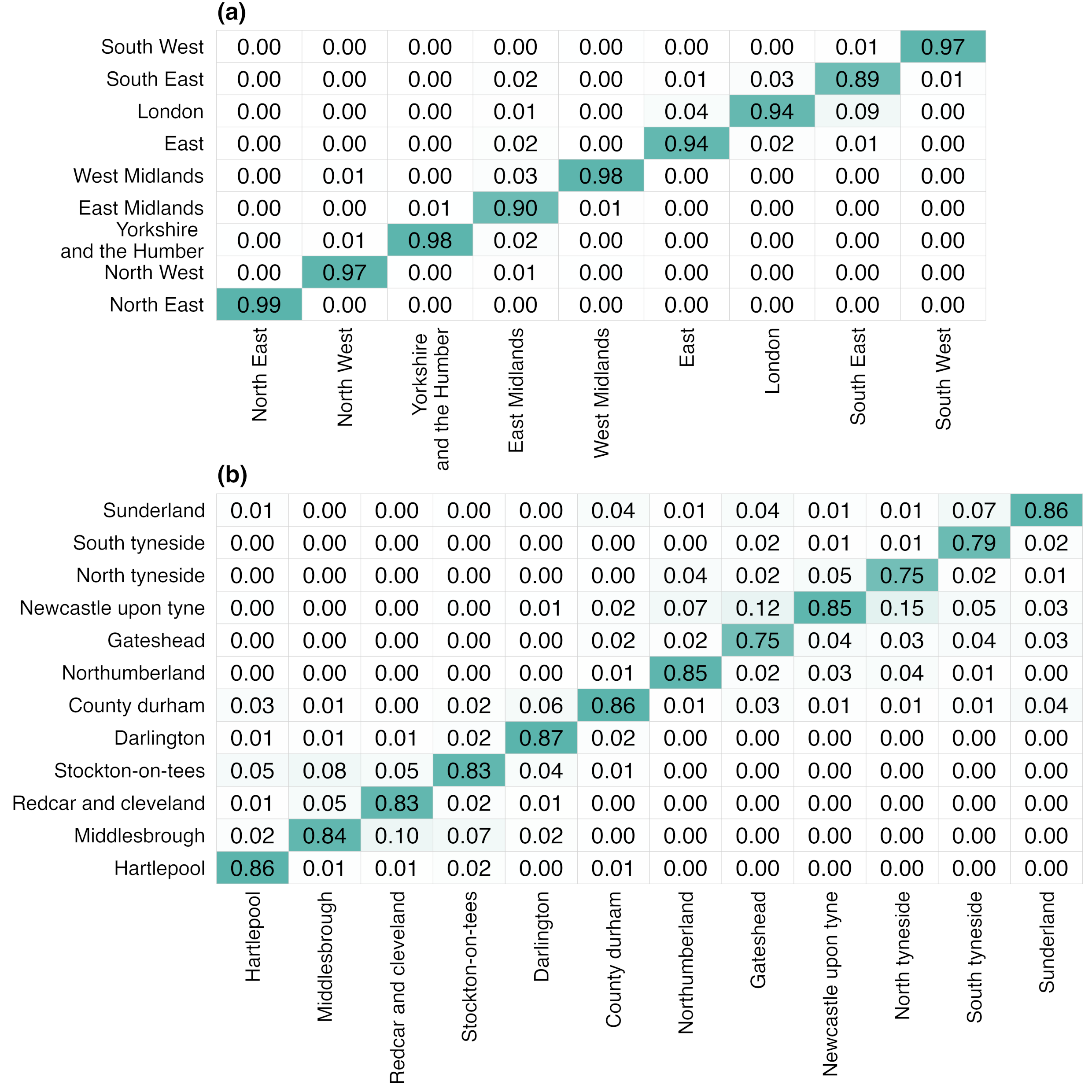

Supplement: S3 Fig — In this figure, the mobility matrices illustrate the commuting patterns across the regions of England (see S3a Fig) and Lower Tier Local Authorities (LTLAs) within the North East region (see S3b Fig). Each column in the matrix represents the fraction of a region’s (or LTLA’s) population that commutes to other regions (LTLAs), signifying the outflow from the corresponding region (LTLA) indicated in the figure. On the other hand, the rows depict the inflow to any given region (LTLA) from all other regions (LTLAs). Notably, the diagonal elements (indicated by green box) represent the fraction of the non-commuting population, those who remain within their own region (LTLA). As each column reflects the distribution of a region’s (LTLA’s) population across all destinations, the sum of each column is equal to one. In S3a Fig, the mobility data for the regions of England reveals that a significant majority of the population does not commute to other regions on a daily basis for work or other purposes. This may be the reason for the lack of observable impact at this broader spatial resolution. In contrast, S3b Fig details a substantial number of commuters among LTLAs within the North East region of England. The data indicates that approximately 15% to 25% of individuals are commuting to other LTLAs, which appears to significantly influence transmission dynamics. (TIFF) [file pcbi.1013642.s003.tif]

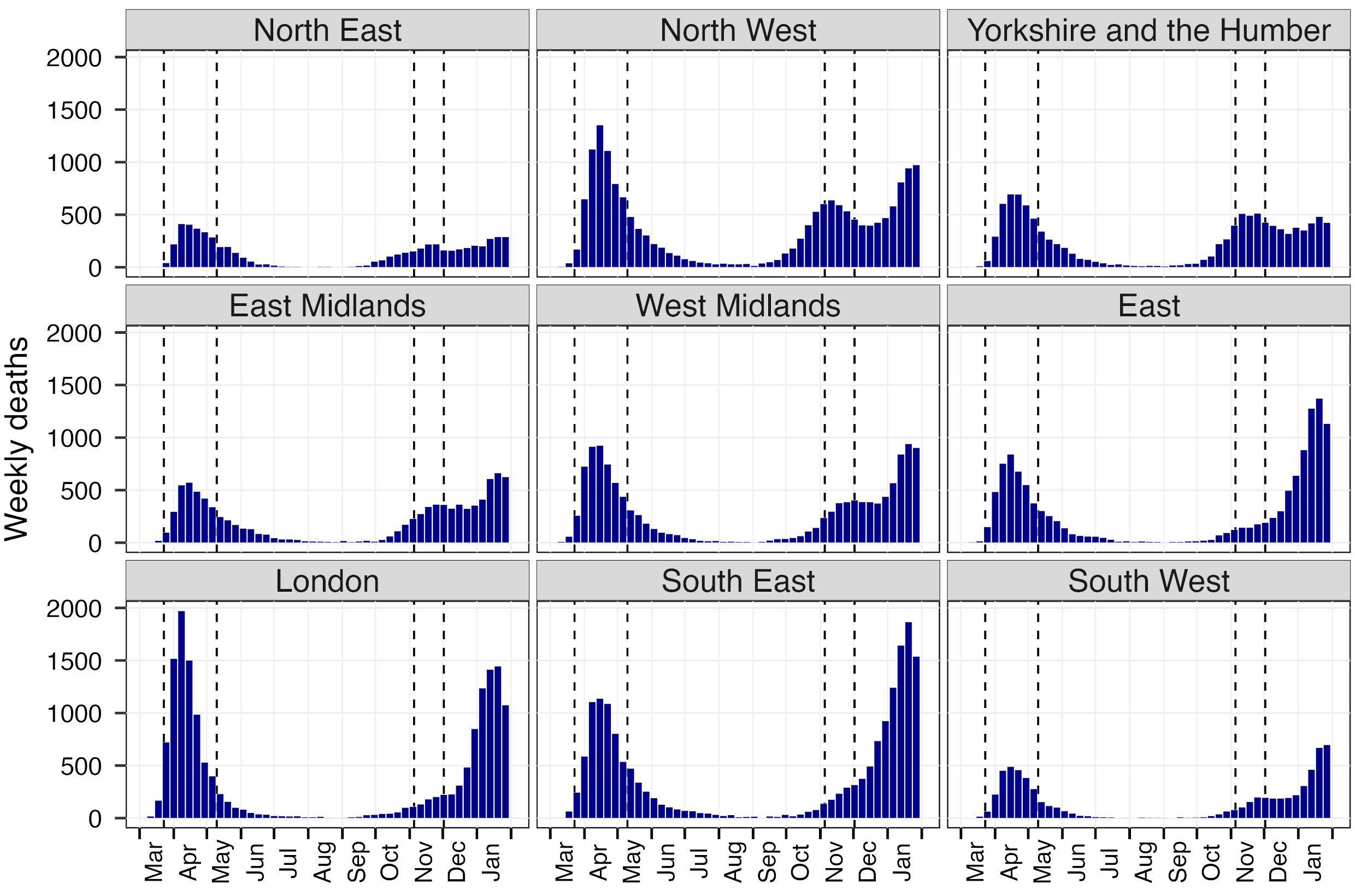

Supplement: S4 Fig — Vertical lines show the lockdown periods in England. (TIFF) [file pcbi.1013642.s004.tif]

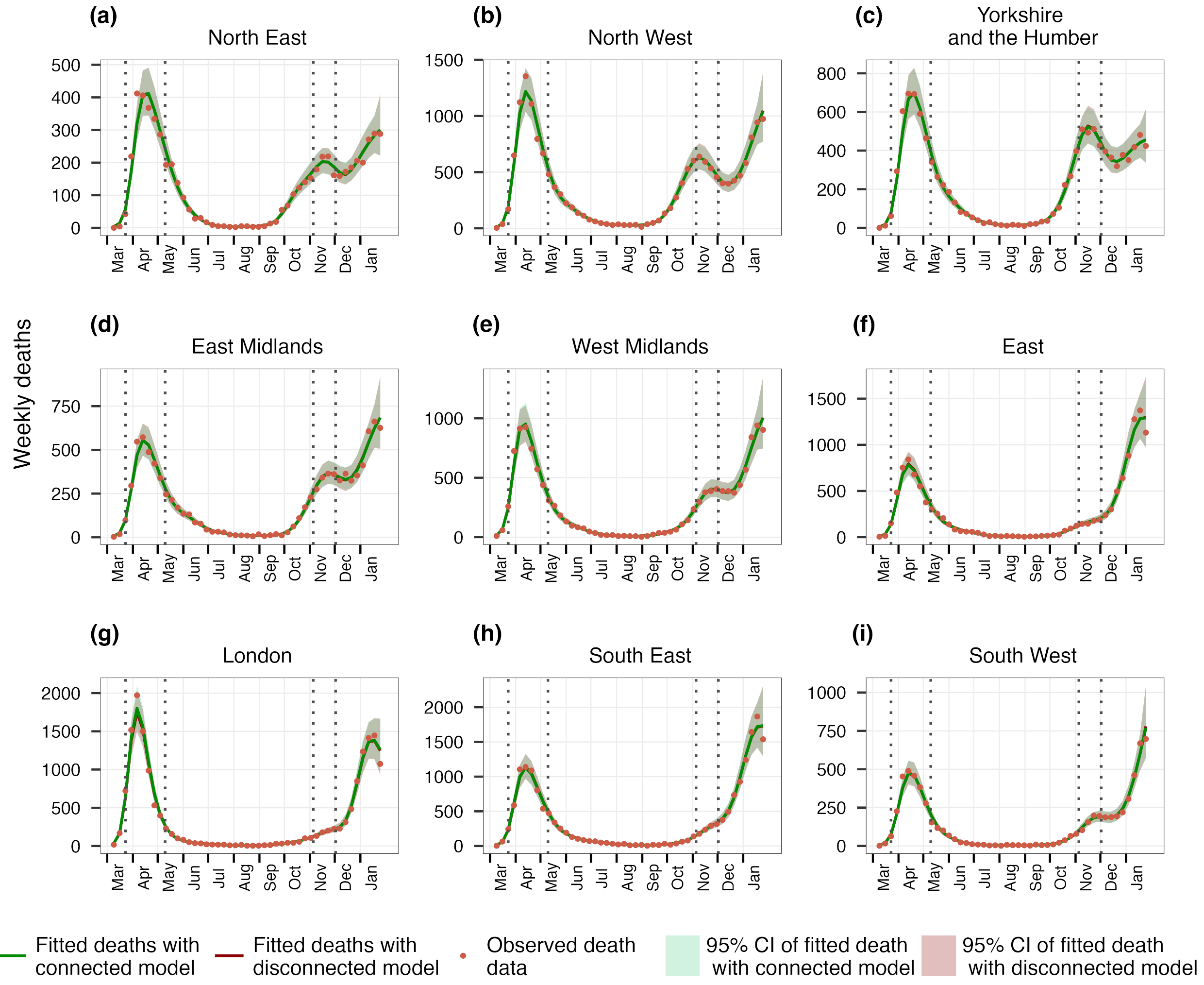

Supplement: S5 Fig — The green (red) curve is the fitting with the connected (disconnected) model and 95% credible intervals are shown around the curve. Orange dots are the weekly deaths observed for each region. Dotted vertical lines show the lockdown periods. (TIFF) [file pcbi.1013642.s005.tif]

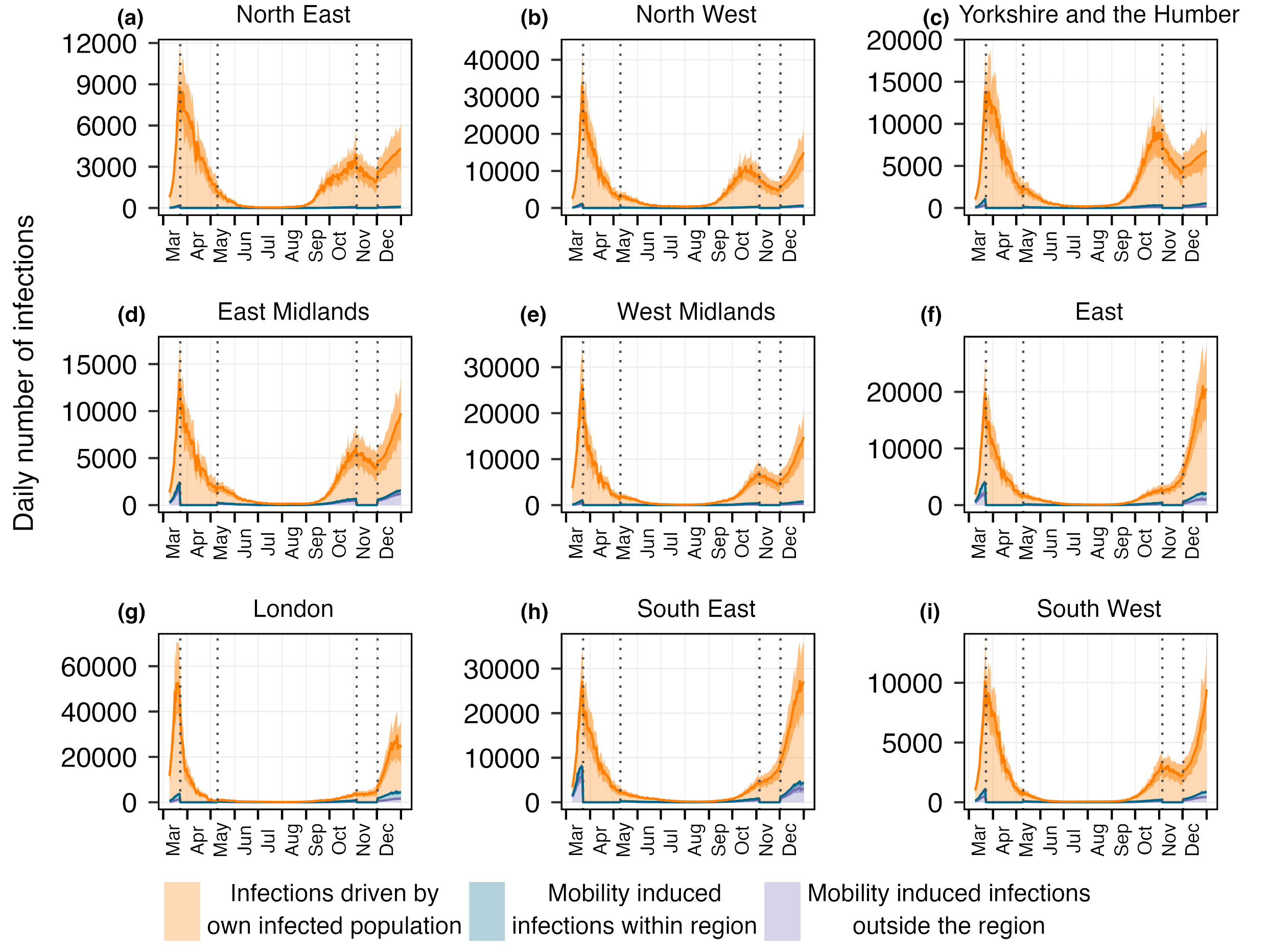

Supplement: S6 Fig — The colored segments illustrate the contributions from each source: orange indicates infections generated by the own infected population within their own region, blue denotes infections acquired locally due to visits of infected individuals from other regions, and purple represents infections contracted outside the region as a result of travel. Vertical lines show the lockdown periods. (TIFF) [file pcbi.1013642.s006.tif]

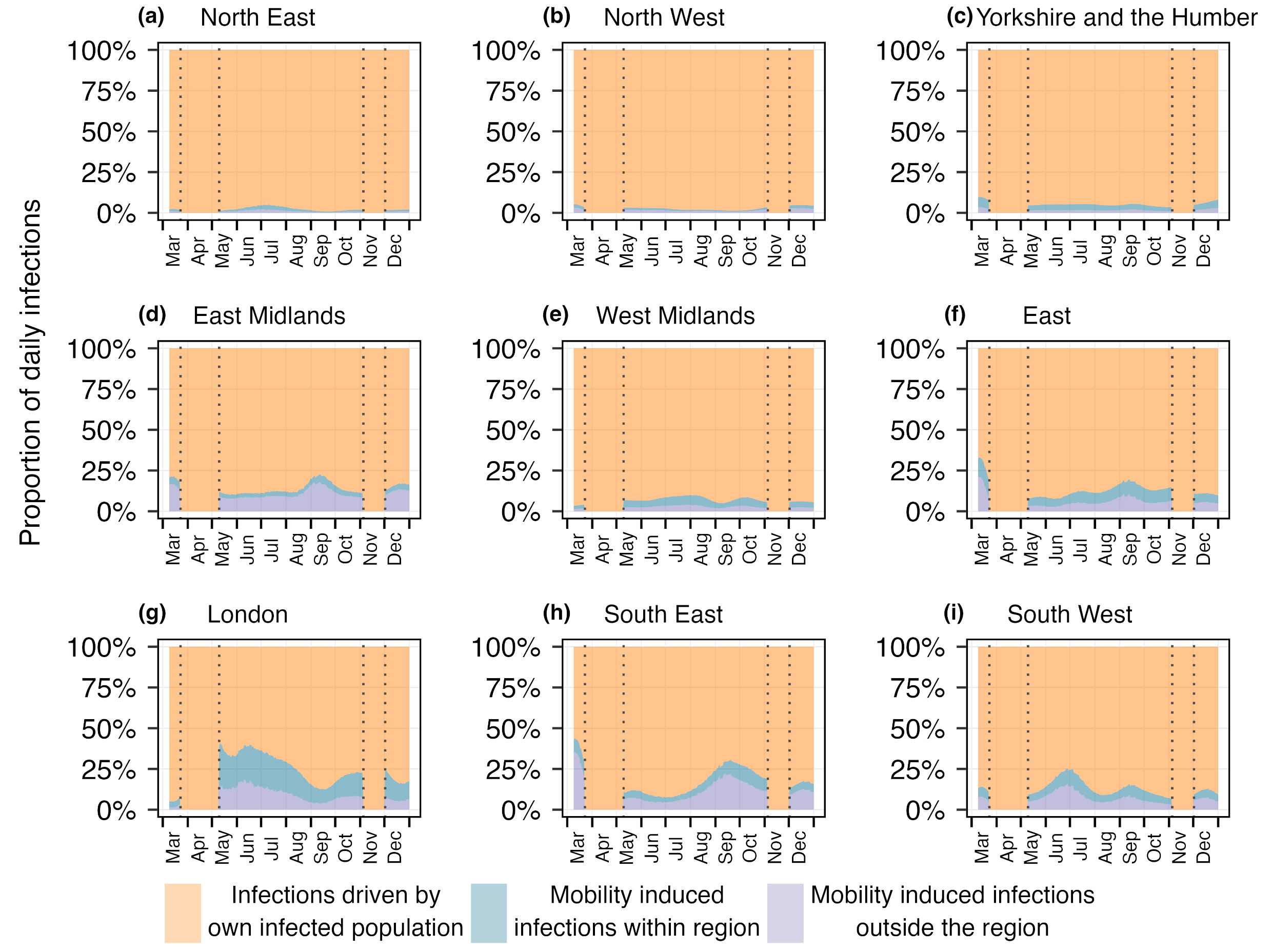

Supplement: S7 Fig — The proportion of daily infections coming from three different sources and vertical dotted lines represent the lockdown periods. (TIFF) [file pcbi.1013642.s007.tif]

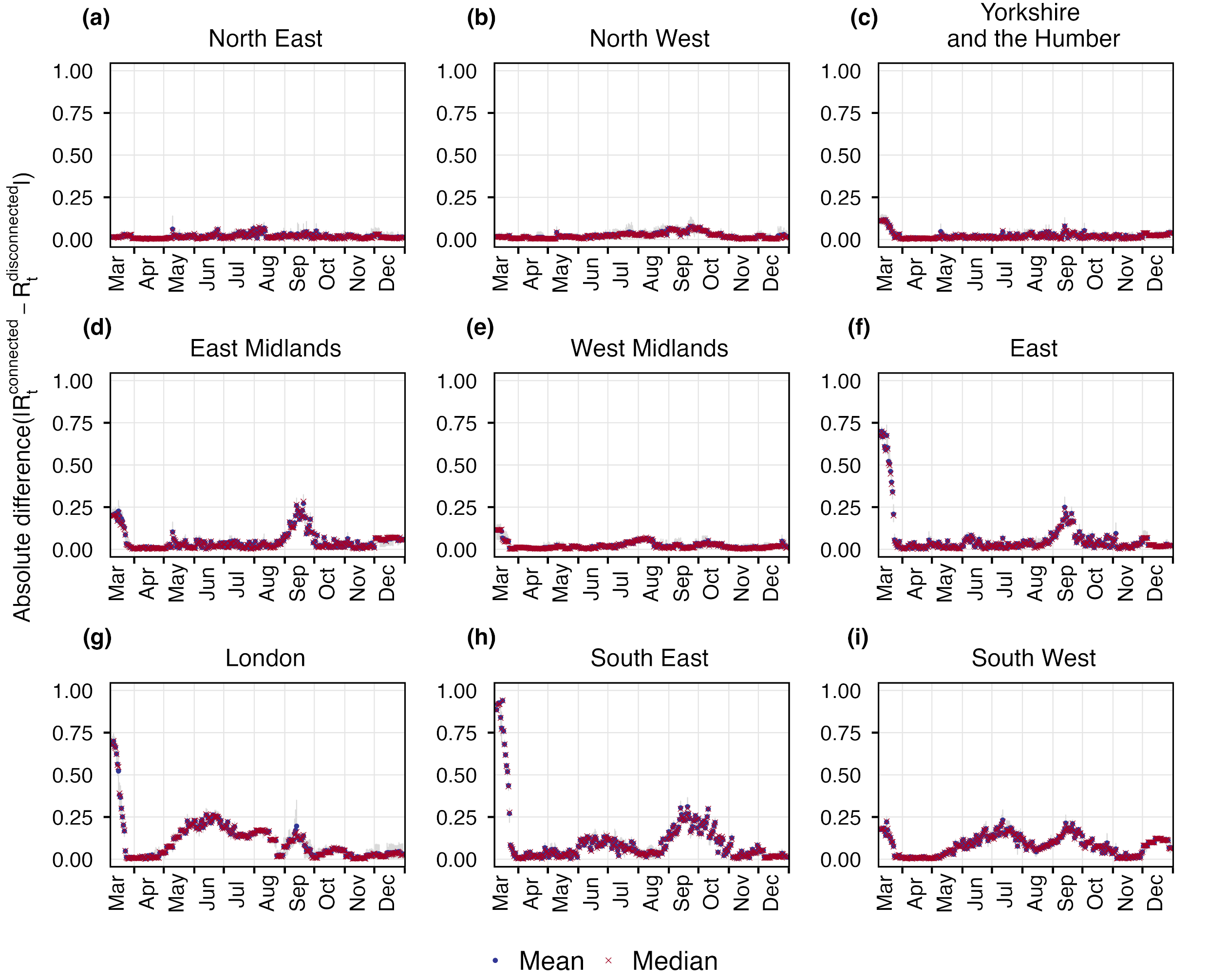

Supplement: S8 Fig — Blue dots and red crosses show the mean and median of the absolute differences at each time point. Grey bars show the corresponding 95% credible intervals. (TIFF) [file pcbi.1013642.s008.tif]

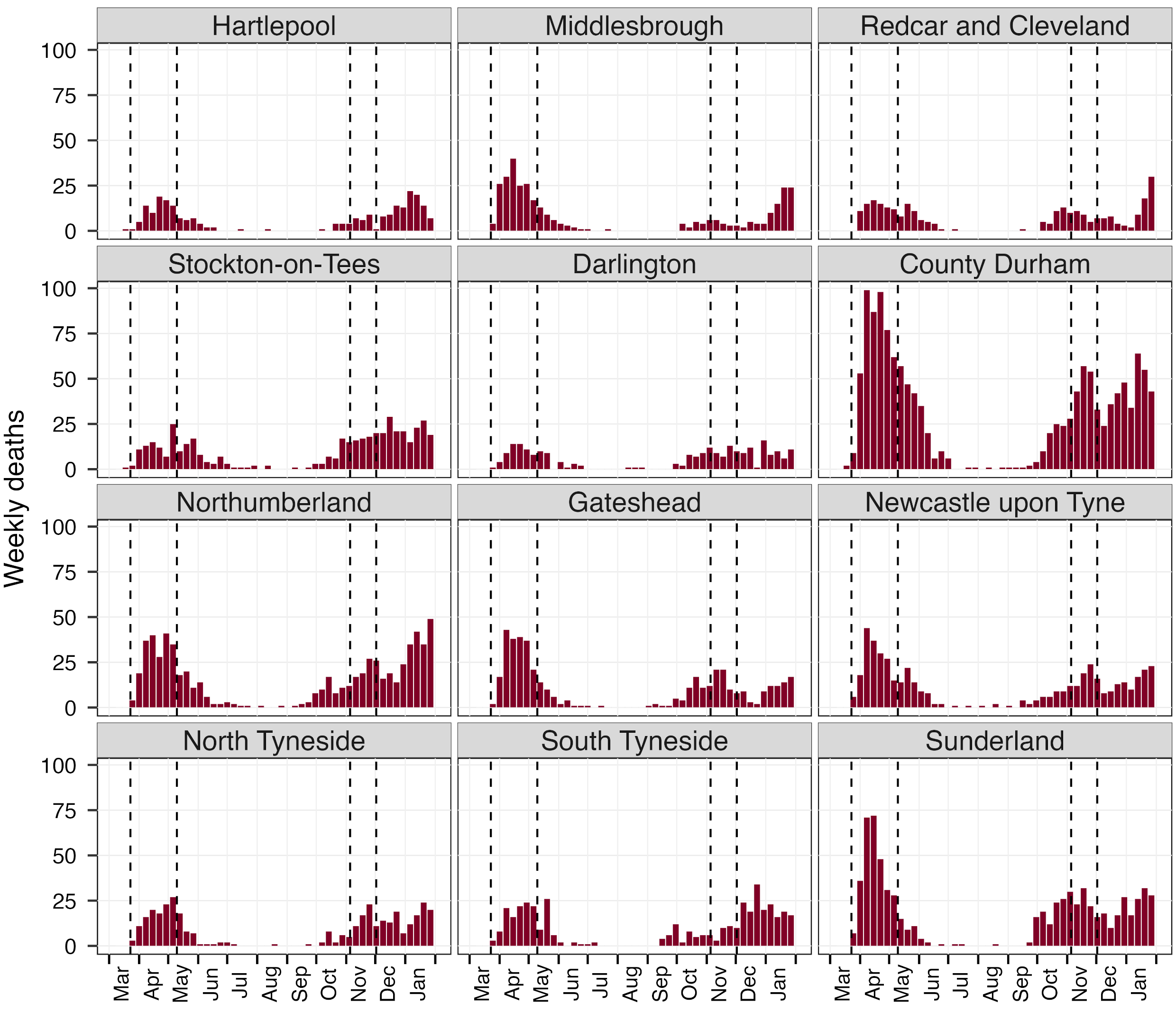

Supplement: S9 Fig — Vertical lines show the lockdown periods in England. (TIFF) [file pcbi.1013642.s009.tif]

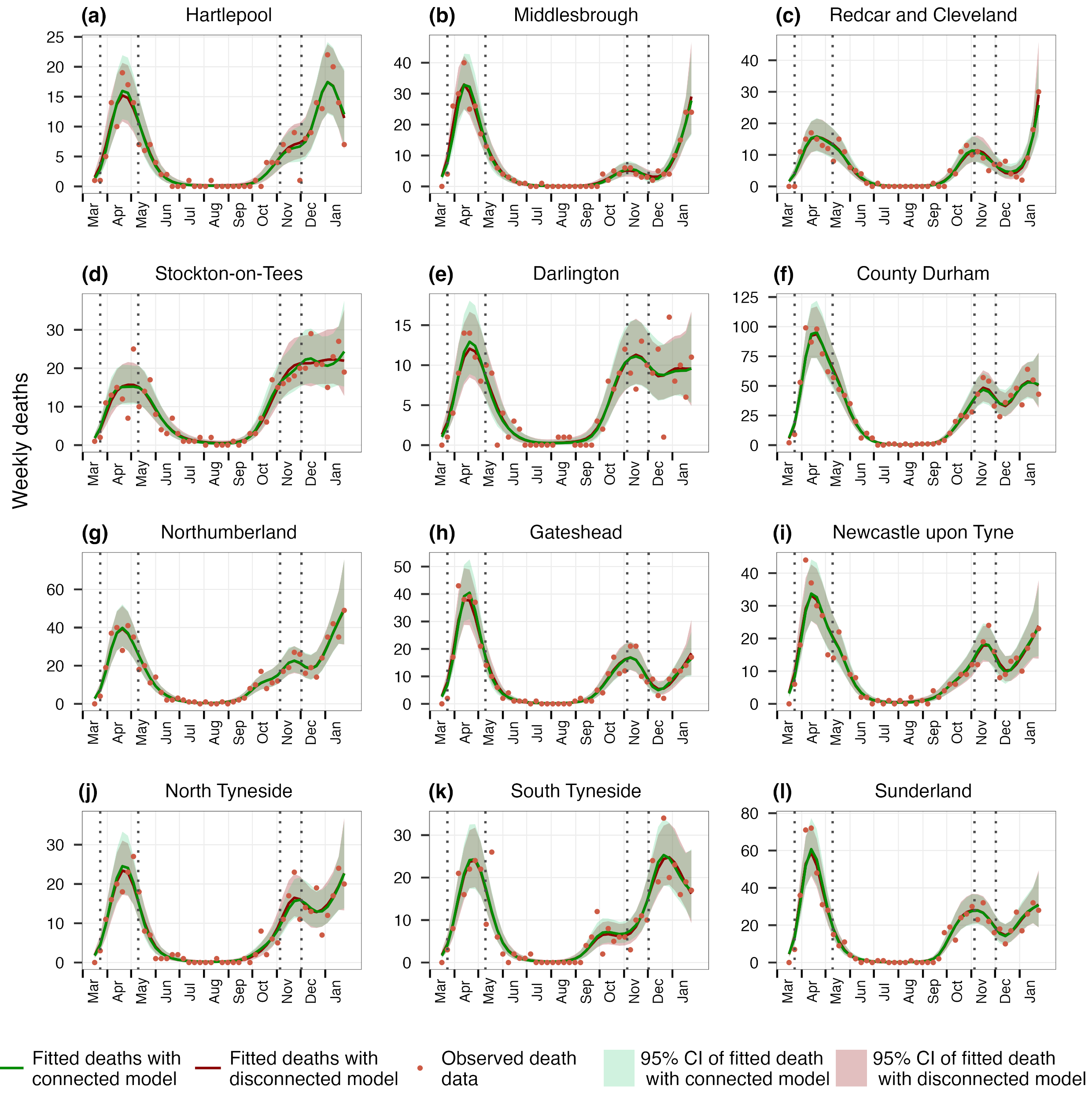

Supplement: S10 Fig — The green (red) curve is the fitting with the connected (disconnected) model and 95% credible intervals are shown around the curve. Orange dots are weekly deaths observed for each LTLA. Dotted vertical lines show the lockdown periods. (TIFF) [file pcbi.1013642.s010.tif]

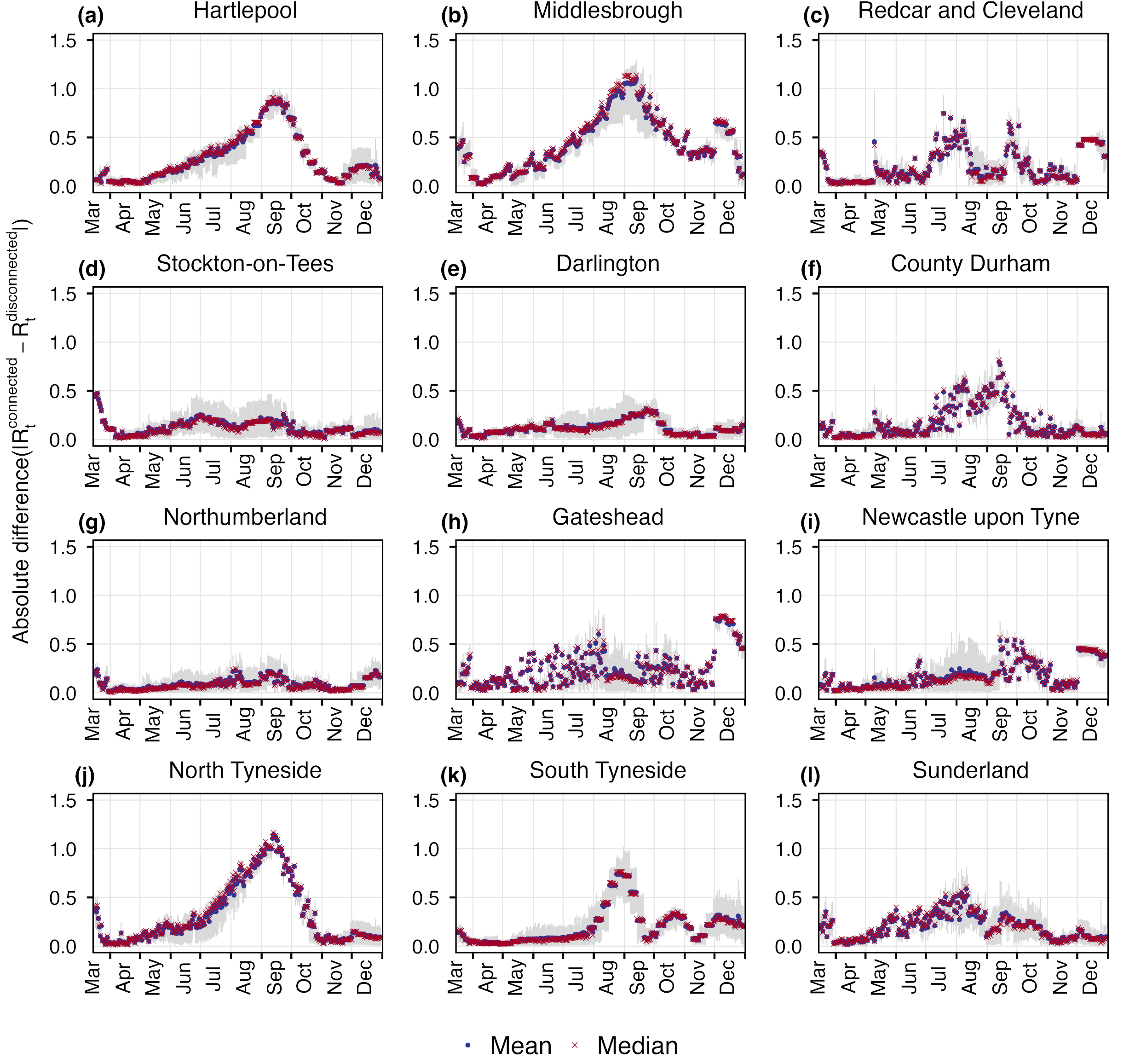

Supplement: S11 Fig — Blue dots and red crosses denote the mean and median of the absolute differences at each time point. Grey bars show the corresponding 95% credible intervals. (TIFF) [file pcbi.1013642.s011.tif]

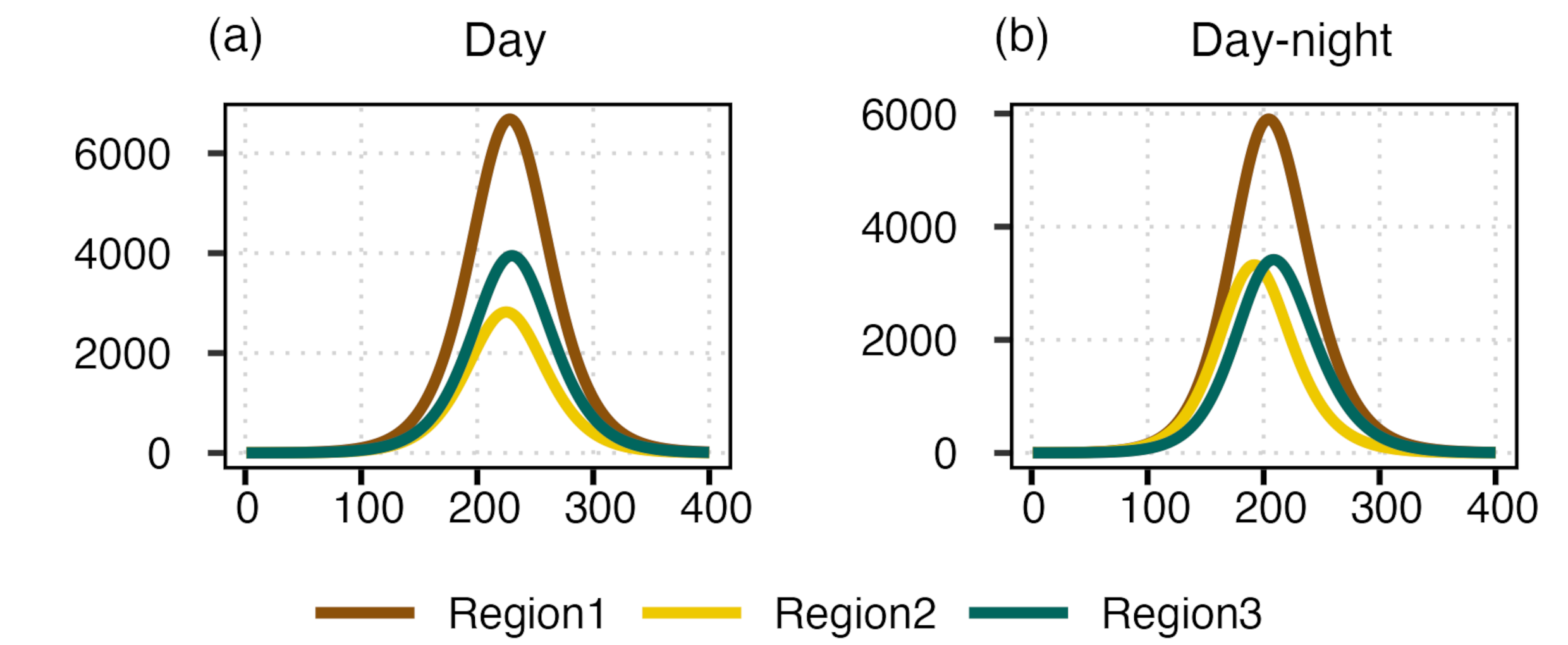

Supplement: S12 Fig — We consider three regions with populations of 15,00,000, 5,00,000, 10,00,000, respectively. The reproduction numbers are Rt(1)=1.1, Rt(2)=1.7, Rt(3)=1.1, where Rt(i) represents the reproduction number at time t for region i. The mobility matrices are considered as follows:Cday=[Region1Region 2Region 3Region 10.70.070.21Region 20.250.850.09Region 30.050.080.7]Cnight=[Region1Region 2Region 3Region 1100Region 2010Region 3001]where, Cij denotes the fraction of population of region j commute to region i. In S12a Fig, we consider a daily time-scale, where the mobility matrix is represented by Cday and remains constant throughout the unit time (a full day). In S12b Fig, we consider a finer time scale as half-day intervals. For the first half of the day the mobility matrix is considered as Cday and for the second half of the day the mobility matrix is Cnight to reflect that people typically return to their home regions at night. The generation time distribution is also adjusted accordingly. Here, Rt(2)>Rt(1),Rt(3) and therefore, in S12b Fig, when population spends half of the day in their home regions, infections increase in region 2 and decrease in region 1 and region 3 in comparison to S12a Fig. This shows the flexibility of this framework in adapting different time scales. (TIFF) [file pcbi.1013642.s012.tif]

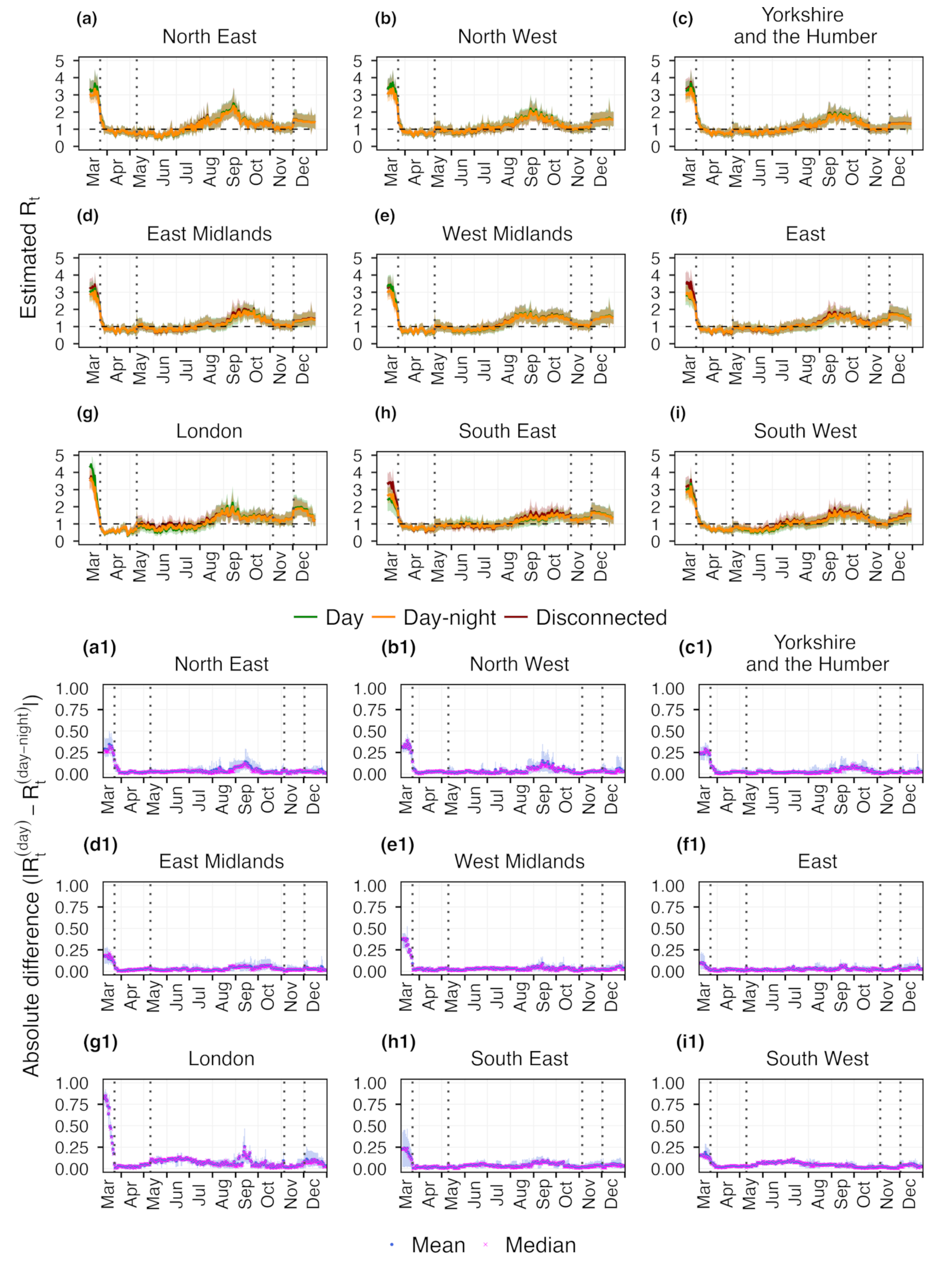

Supplement: S13 Fig — We consider two time scales: “day" (same as connected Rt as in Fig 4 in the main text), which are represented by the green curves in S13a-i Fig, and “day-night" where unit time is half of a day and represented by the orange curves, and disconnected Rts are shown by the red curves. In the “day-night" case, the first half of a day uses the mobility matrix as shown in S3a Fig, and for the second half we consider the identity matrix to account for the fact that people return to their home region at night. No such difference appears in the results based on the time-scale. We quantify this by calculating absolute residuals |Rt,i(day)−Rt,i(day−night)| between green and orange curves (see S13a1-i1 Fig). Here, blue dots and purple crosses are the mean and median of the residual distribution at each time point, respectively. The corresponding error bars are represented in light blue color. (TIFF) [file pcbi.1013642.s013.tif]

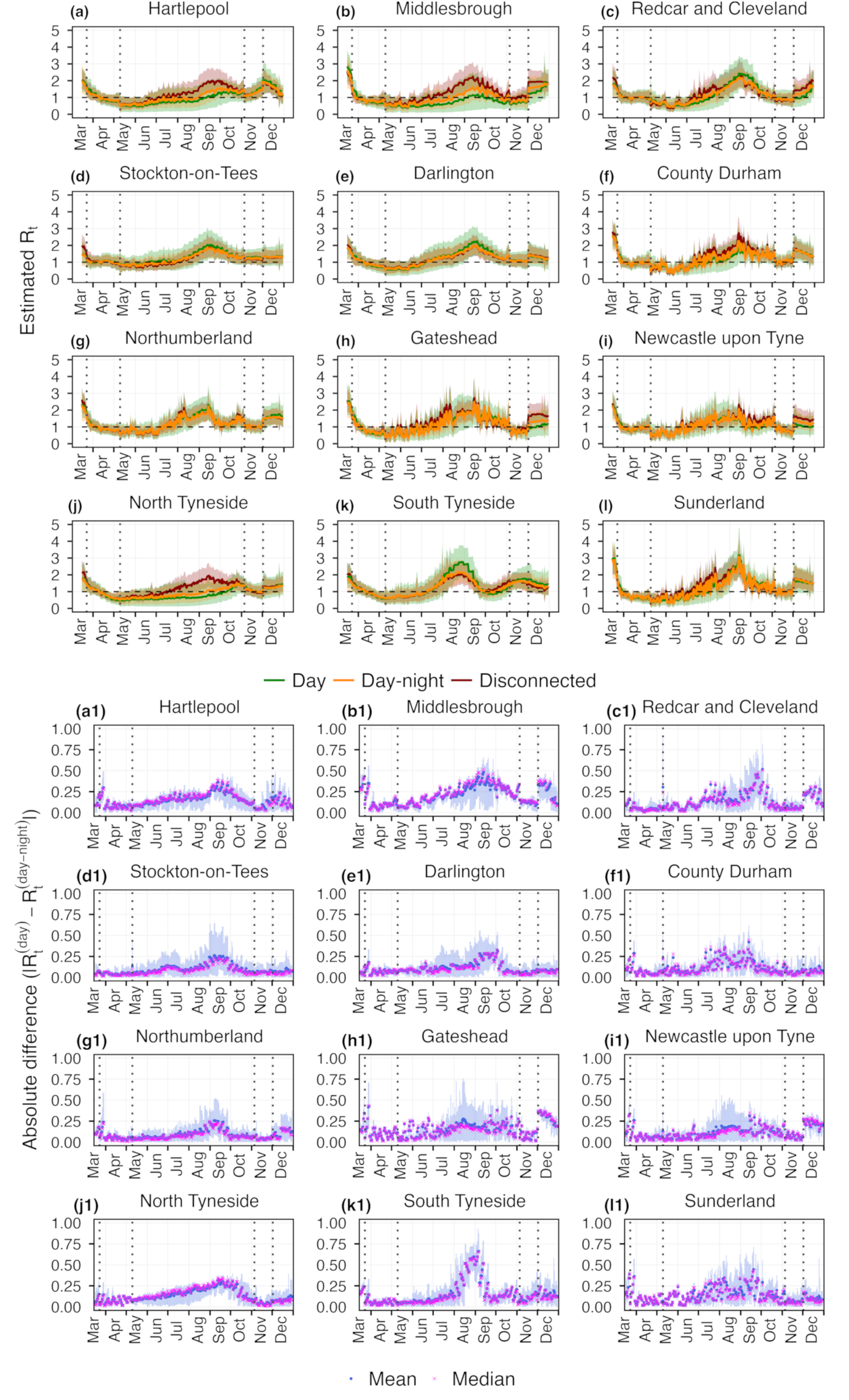

Supplement: S14 Fig — We consider two time scales: “day" (same as connected Rt as in Fig 5 in the main text), which are represented by the green curves in S14a-l Fig, “day-night", where unit time is half of a day and represented by the orange curves, and disconnected Rts are shown by the red curves. In the “day-night" case, the first half of a day uses the mobility matrix as shown in S3b Fig, and for the second half we consider the identity matrix to account for the fact that people return to their home region at night. We quantify this by calculating absolute residuals |Rt,i(day)−Rt,i(day−night)| between green and orange curves (see S14a1-l1 Fig). Here, blue dots and purple crosses are the mean and median of the residual distribution at each time point, respectively. The corresponding error bars are represented in light blue color. Therefore, in general results obtained using daily time scale seem robust when comparing them to a potentially more realistic time scale (day-night). (TIFF) [file pcbi.1013642.s014.tif]

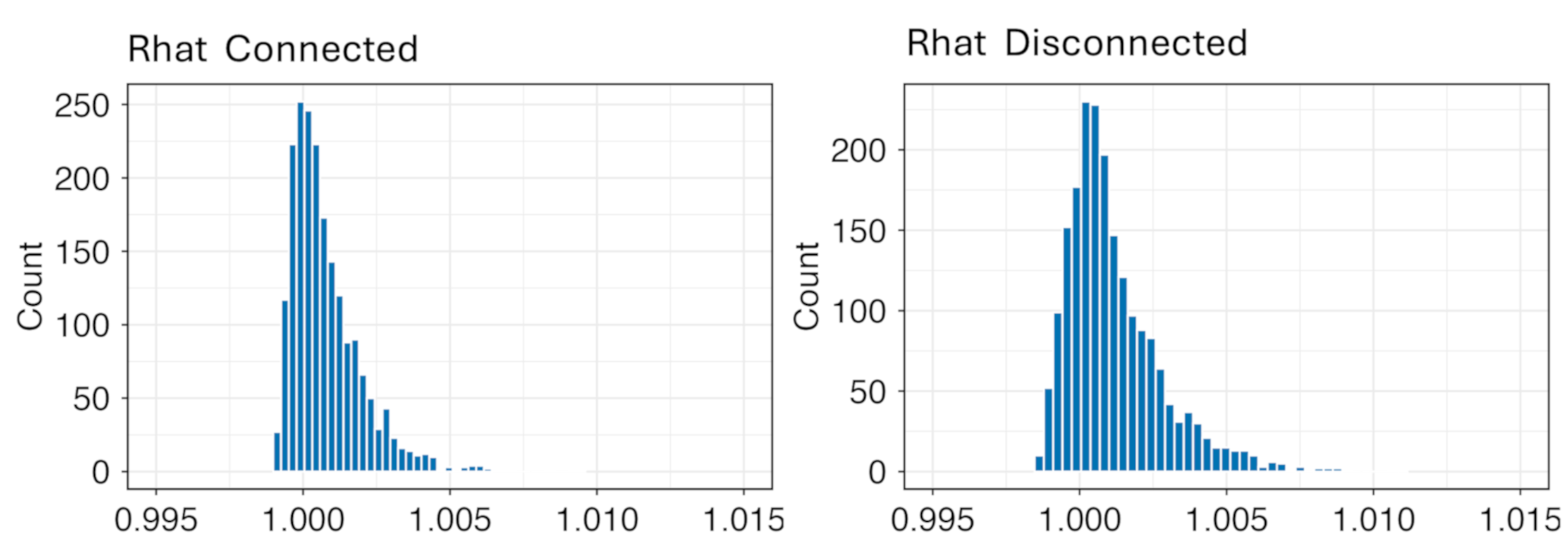

Supplement: S15 Fig — Values less than 1.01 indicate MCMC convergence. (TIFF) [file pcbi.1013642.s015.tif]
