# Supplementary material for: Incorporating human mobility to enhance epidemic response and estimate real-time reproduction numbers
Source: PLoS Comput Biol. 2025 Nov 13;21(11):e1013642. doi: 10.1371/journal.pcbi.1013642 (PMC12614531; doi:10.1371/journal.pcbi.1013642)
Supplement: Attachment — Submitted filename: roy_pcb_review.pdf [file pcbi.1013642.s016.pdf]

Review of “Incorporating human mobility to enhance epidemic response and estimate real-time reproduction numbers” by Roy et al.

This article proposes an extension of the renewal equation approach for Rt estimation that includes the effect of commuting mobility. The proposed framework is first validated in a controlled environment using synthetic data, and then applied to a real-world case study considering reported death data from 2020 COVID-19 waves in England.

Overall, my review for this article is positive. Reliable Rt estimates are of clear importance especially when facing emerging outbreak such as COVID-19, and tools that allow corrections for the impact of human mobility are important and needed. However, in the current status the paper is missing key information on the analysis and proper quantitative justifications of the main claims. These issues can be nonetheless approached with a review of the manuscript as they do not constitute fundamental flaws in the proposed approach to my judgement.

Please find below my comments aimed at improving the presentation and the justification of the findings.

Introduction

1. The justification for the need for the proposed approach appears weak. The message that emerges is that mobility is important when modeling the transmission of infectious diseases, but no further explanation and/or examples is provided. Further details on how mobility (especially commuting, which is the type of mobility considered in the paper) can affect the spread (and as a result the estimation of Rt) would significantly strengthen the support for the proposed approach. My feeling is that the current justification for the need of the inclusion of mobility in the renewal equation approach is too shallow and with little effort it can be given more depth
2. The comparison with other modeling approaches lacks a fundamental class: metapopulation models. Metapopulation models explicitly includes a spatial component as well as mobility, therefore it seems natural to me to mention them, with strengths, limitations, and comparison with the renewal equation approach
3. An entire paragraph is dedicated to the discussion of the availability of mobility data as well as models of human mobility. This seems misplaced to me. Indeed, the main contribution of this paper is methodological, therefore I do not see the need to include these practical considerations in the introduction. They may be instead relevant for the discussion and the conclusion, when authors discuss further application and expansion of their framework.

Materials and Methods

1. I think it would be more correct to refer to g as the generation time distribution (also throughout the text)
2. The integral definition of gs needs some more explanation for the extremes of the integral

3. Line 110, when introducing the mobility matrix m, I suggest also specifying how to interpret the diagonal element (i.e., the fraction of non-commuting individuals), otherwise some readers may assume that mii = 0 only to find out later that this is not the case.
4. In the simulated data generation section, it is not clear which values of Rt have been used and how they have been chosen
5. In line 203 R0 is introduced for the first time. I understand that this is R(t=0), but given the epidemiological importance of this quantity I suggest to spend a couple of words to explicitly say what R0 is in this context
6. Given how central mobility is, I suggest providing more details on the mobility matrices used in the England case study (from where they are taken, how data is collected, which year, etc). Lines 215-217
7. The use of mobility data from the Google mobility report to modulate transmission should be justified and supported with references, see for example: https://www.pnas.org/doi/10.1073/pnas.2421993122, https://www.nature.com/articles/s42254-021-00407-1
8. The functional form of Rt (~line 226) is never justified. What is the rationale behind this choice? Is this a standard approach (if so, relevant references should be added)? Did you perform any sensitivity analysis to assess the impact of this choice?
9. Details on model fitting procedure are completely missing. Relevant information on model calibration should be added in both the main text and if needed in the Supplementary Information

Results

Overall, the comparisons between estimated Rt are at the moment purely visual, qualitative, and anecdotal. I believe that with relatively low efforts the comparisons can be also made in quantitative terms and as result solidify the narrative

1. Figure 3a/b/c. The deviation between the red and the green curves can be better characterized, at the moment it is purely visual and not quantitative. A possible solution would be to compute correlations between one-step increments in Rt (i.e., (Rt) - (Rt-1)) of green and blue curves (e.g., ground truth vs connected Rt) and of blue and red curves (e.g., ground truth vs disconnected Rt). I expect higher correlations between one-step increments of green and blue curves, and this would further support the claim that the connected Rt provides a better estimate of the ground truth.
2. Similarly, in lines 288-314 there is a long discussion on the impact on mobility which is only supported by visual/anecdotal evidence. A possibility to make the discussion more quantitative can be the following. You can correlate, in each region, the (absolute) residuals of the red curve with respect to the ground truth (i.e., the error made by the disconnected model) with the percentage of new infections induced by mobility for that region (i.e., the information in panels 3g,h,i). I expect positive correlations, suggesting that the disconnected model makes most of the errors when the contribution to infections due to mobility is higher.
3. Figure4 and Figure 5. It would be beneficial to show (either within the plot or in the SI) the (absolute) residuals between green and red curves, in order to be able to actually quantify the difference between the two estimated Rts. Mean, medians, and

other statistics of these residuals can be mentioned when authors comment on the small difference between the curves.

Discussion/Conclusions

1. The paper concludes with a discussion on computational resources and further direction in this sense. I find this unnecessary, especially because information about computational intensiveness of the proposed approach is never discussed in the paper, without setting a baseline for future developments.

Other comments:

1. I apologise if I missed them, but references to the data used are completely missing, namely death data from England, population of the regions study, mobility matrices, citation to the Google mobility report data. All these references should be opportunely cited.
2. The manuscript would benefit from a thorough proof-read as there are some repetitions, typos, incorrections. Some examples
    - 55-59, these sentences repeat the same concept twice
    - 159: should be “detailed”
    - Equation 4 is missing top extreme of the summation in k
    - Title of section 2.3, there’s a “:” in the title
    - Line 334: I would suggest avoiding the term insignificant as no statistical test has been performed
    - Line 374, “reductive factor” of what?
    - Line 381, avoid using etc.
    - Line 382, SARS-CoV-2 (- missing)
