# Supplementary material for: Incorporating human mobility to enhance epidemic response and estimate real-time reproduction numbers
Source: PLoS Comput Biol. 2025 Nov 13;21(11):e1013642. doi: 10.1371/journal.pcbi.1013642 (PMC12614531; doi:10.1371/journal.pcbi.1013642)
Supplement: Attachment — Submitted filename: Response to Reviewers.pdf [file pcbi.1013642.s017.pdf]

To
The Academic Editor and Section Editor,
PLOS Computational Biology

We convey our personal thanks for your email regarding our manuscript **Incorporating human mobility to enhance epidemic response and estimate real-time reproduction numbers (PCOMPBIOL-D-25-00829)** on 24.06.2025 and the attached reports of the referees. Our sincere thanks to the reviewers for their appreciation as they found “the study interesting” and mentioned “Overall, my review for this article is positive”. We find the comments of the referees are very constructive and extremely useful to improve the previous version of the manuscript. We have addressed all the points and incorporated all the suggestions by the referees. We have made the corrections in **blue** color in the revised manuscript. We are hopeful that the improved version will be considered for further evaluation.

We look forward to your positive decision.

Sincerely,
Mousumi Roy, on behalf of all co-authors

We have addressed all the comments by the referees. For a detailed discussion, please refer to the response to Referees. We show all the comments in **red,** responses in **black,** and all the changes to the revised manuscript and SI are highlighted in **blue**.

Response to Referees

Reviewer #1:

This work presents a new mathematical approach including mobility between regions to the computation of the net reproduction number. It presents results from a simulation study and from applying the methodology to real data from SARS-CoV-2 in England.
Overall, I found the study interesting. The new equation proposed is quite straightforward and easy to understand, and at the same time it aims to tackle the mobility issue in the computation of the transmissibility in a certain area, which I personally did not see before.

Response: Thank you so much for finding the study interesting. We have addressed all the suggestions and updated the revised manuscript accordingly. Point wise all the answers of the comments are listed below:

Major suggestion:

1.However, this study doesn't take into account the fact that people potentially do not spend all day in another region different from the one where their residence is. An option would be to divide the day in segments in which the matrix "m" changes. Perhaps it would be interesting to see how much this affects the results. Also / Alternatively, it would be nice to justify how your model somehow can account for the fact that the presence of some individuals in other regions only lasts a fraction of the day (week).

Response: Thank you so much for the comment. To address this point, we simulate two scenarios with three regions, considering two different time scales keeping all the other parameters fixed. In the following figure R1(a), the timescale is "Day" and R1(b) represents the time scale as half of the day which is shown as "Day-night". We plotted the daily infection over time for the three regions based on the two scenarios. This shows the flexibility of this framework in adapting different time scales.

We added this to the **SI (Fig L. on page 14)** and described as follows:

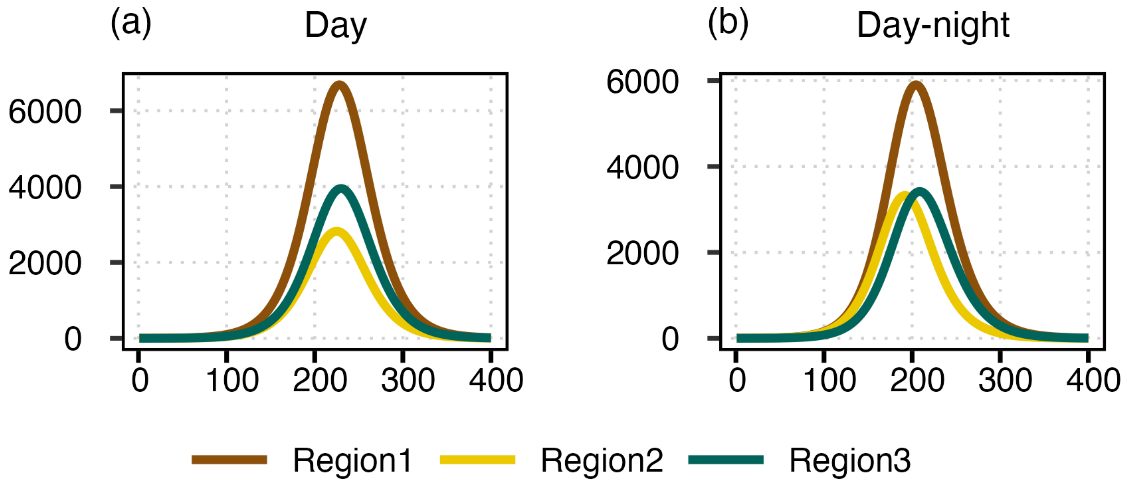

R1: Flexibility of the framework in adapting different time-scales. We consider three regions with populations of 15,00,000, 5,00,000, 10,00,000, respectively. The reproduction numbers are $R_t^{(1)} = 1.1$, $R_t^{(2)} = 1.7$, $R_t^{(3)} = 1.1$ where $R_t^{(i)}$ represents the reproduction number at time t for region i. The mobility matrices are considered as follows:

$C_{day} =$

0.7	0.07	0.21

0.25	0.85	0.09
0.05	0.08	0.7

$C_{night} =$

1	0	0
0	1	0
0	0	1

where, C_{ij} denotes the fraction of population of region j commute to region i.
In Fig.R1(a), we consider a daily time-scale, where the mobility matrix is represented by C_{day} and remains constant throughout the unit time (a full day). In Fig.R1(b), we consider a small time scale as half-day intervals. For the first half of the day the mobility matrix is considered as C_{day} and for the second half of the day the mobility matrix is C_{night} to reflect that people typically return to their home regions at night. The generation time distribution is also adjusted accordingly.

Here, $R_t^{(2)} > R_t^{(1)}, R_t^{(3)}$ and therefore, in R1(b), when the population spends half of the day in their home regions, infections increase in region 2 and decrease in region 1 and region 3, in comparison to R1(a).

We also referred to this in the main text on **page 5, lines 145-149** as follows:

In the context of the framework's time scale, if individuals do not spend an entire day in another region, the framework is flexible enough to adopt that fact. A detailed simulation is provided in Fig L in SI. However, in this work, we consider a daily timescale throughout the study, based on the available data.

Therefore, this framework allows any desired time-scale, provided that detailed mobility information is available. Therefore, the model can account for the fact that "**people potentially do not spend all day in another region different from the one where their residence is**". Similarly, if detailed information is available on the average time a person from a region spends outside their home region, our model can also incorporate the fact that "**Presence of some individuals in other regions lasts for a fraction of the day.**" In our work, we do not account for the possibility that individuals may not

spend the entire day in another region, due to the lack of data on the time spent outside the home region in England. If such information is available, our framework is flexible enough to incorporate this information.

However, we consider a scenario with a time scale half-day interval (“day-night”) for regions of England and LTLA’s of North-East region to address the point “**it would be interesting to see how much this affects the results”**.

We consider the mobility matrices used in the main text (described in SI Fig. C(a),(b)) for the first half of the day, for the second half of the day (i.e., night), we use the identity matrix to reflect individuals returning to their home regions. We have also included a quantification by plotting the absolute residuals of estimated R_ts with “Day” and “Day-night” time-scale. **We find there is no core distinction in the obtained results**, **quantified by the absolute difference** $|R_t^{(day)} - R_t^{(day-night)}|$**.** We added this analysis in the SI in **Figs M, N (on pages 15-17)** as follows:

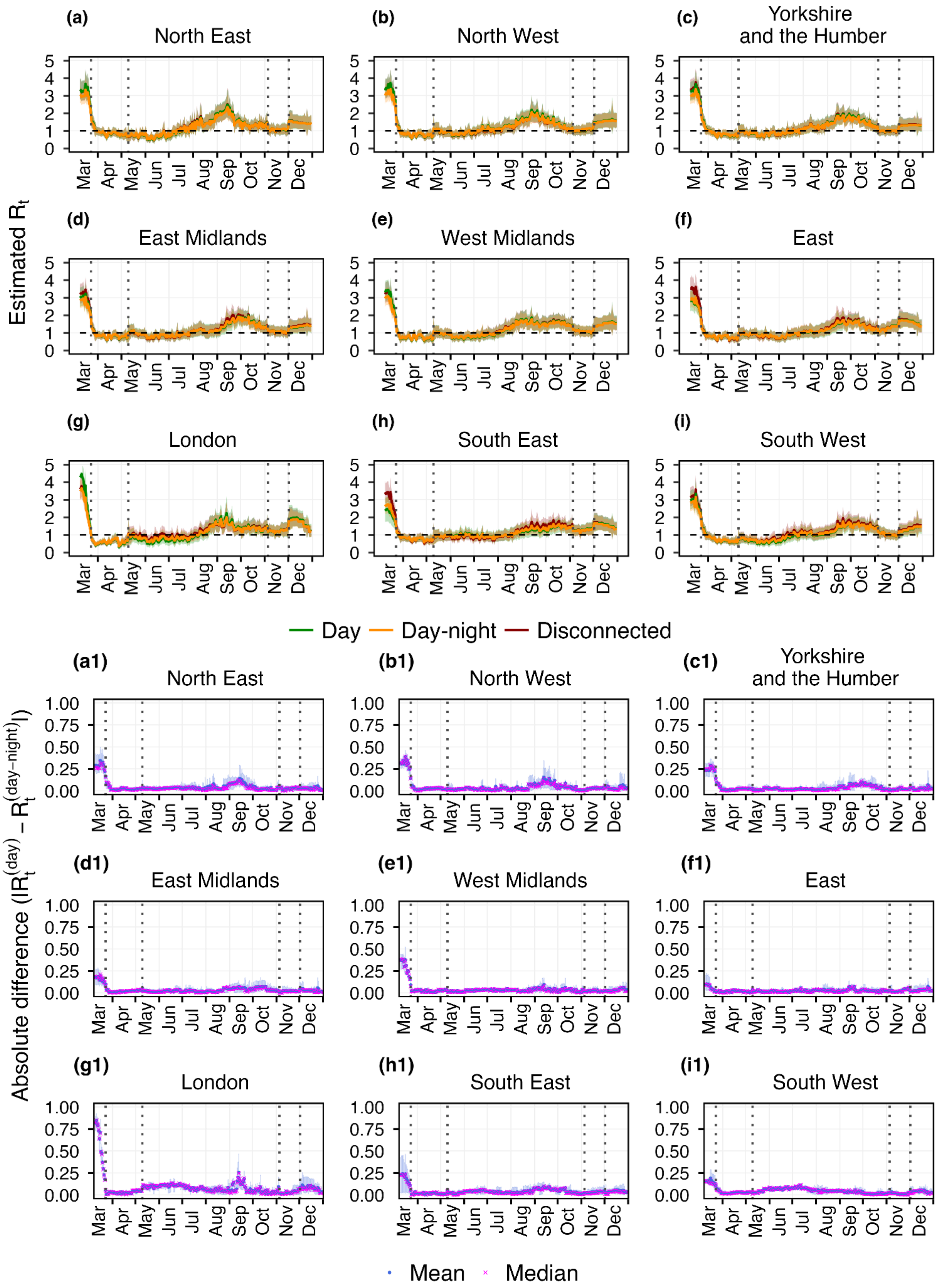

(a) North East
(b) North West
(c) Yorkshire and the Humber
(d) East Midlands
(e) West Midlands
(f) East
(g) London
(h) South East
(i) South West
Estimated R_t
Mar Apr May Jun Jul Aug Sep Oct Nov Dec
Day
Day-night
Disconnected
(a1) North East
(b1) North West
(c1) Yorkshire and the Humber
(d1) East Midlands
(e1) West Midlands
(f1) East
(g1) London
(h1) South East
(i1) South West
Absolute difference ($|R_t^{(day)} - R_t^{(day-night)}|$)
Mean
Median

Fig R2: Effect of time scale on the estimated R_t over the regions of England. We consider two time scales: "day" (same as connected R_t as in Fig.4 in the main text), which are represented by the green curves in R2(a)-(i), "day-night", where unit time is half of a day and represented by the orange curves, and disconnected R_ts are shown by the red curves. In the "day-night" case, the first half of a day uses the mobility matrix as shown in SI Fig B(a), and for the second half we consider the identity matrix to account for the fact that people return to their home region at night. No such difference appears in the results based on the time-scale. We quantify this by calculating absolute residuals $|R_{t,i}^{(day)} - R_{t,i}^{(day-night)}|$ between green and orange curves (see Figs. R2(a1)-(i1)). Here, blue dots and purple crosses are the mean and median of the residual distribution at each time point, respectively. The corresponding error bars are represented in light blue color.

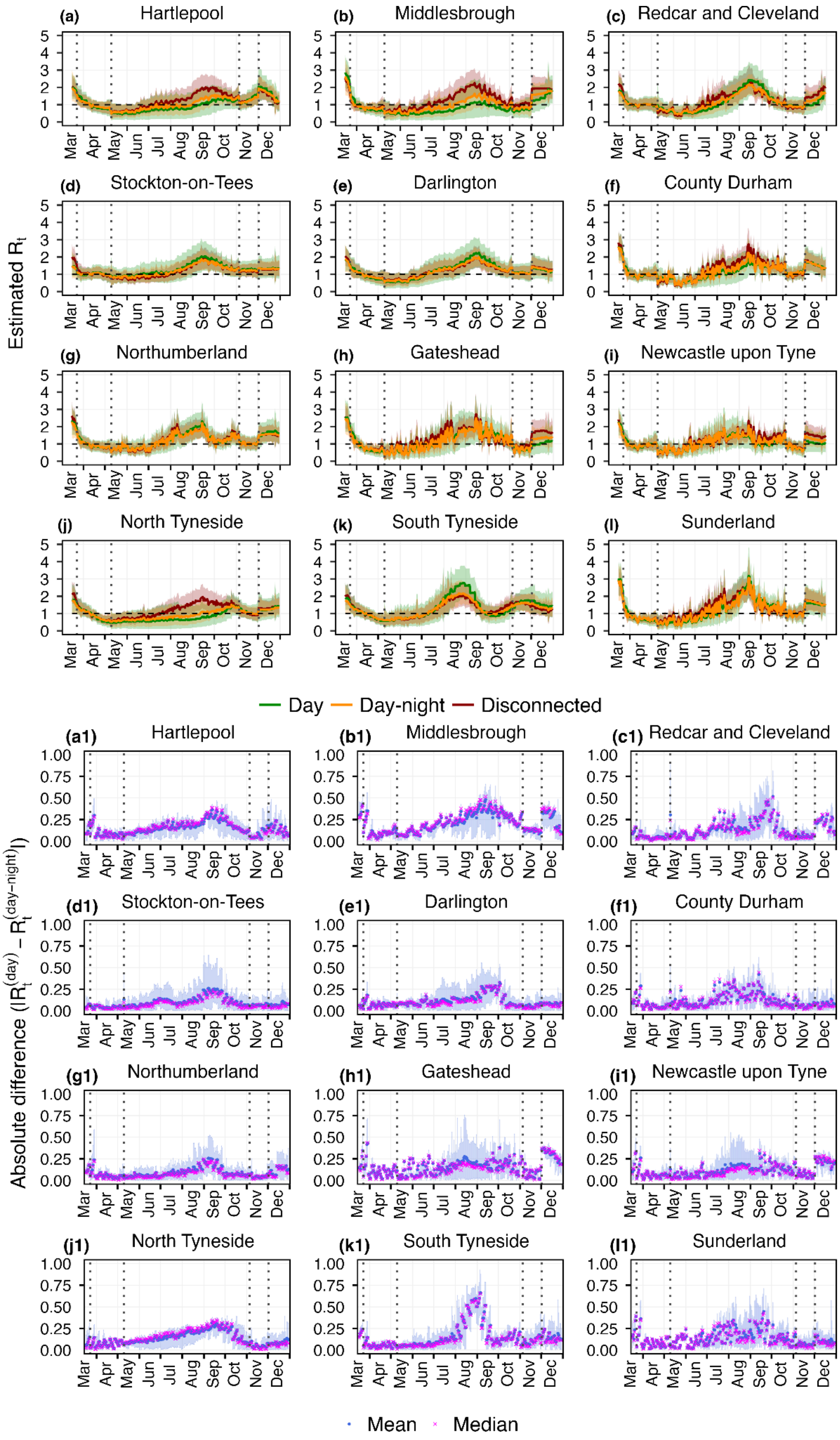

(a) Hartlepool
(b) Middlesbrough
(c) Redcar and Cleveland
(d) Stockton-on-Tees
(e) Darlington
(f) County Durham
(g) Northumberland
(h) Gateshead
(i) Newcastle upon Tyne
(j) North Tyneside
(k) South Tyneside
(l) Sunderland
Estimated R_t
0 1 2 3 4 5
Mar Apr May Jun Jul Aug Sep Oct Nov Dec
Day
Day-night
Disconnected
(a1) Hartlepool
(b1) Middlesbrough
(c1) Redcar and Cleveland
(d1) Stockton-on-Tees
(e1) Darlington
(f1) County Durham
(g1) Northumberland
(h1) Gateshead
(i1) Newcastle upon Tyne
(j1) North Tyneside
(k1) South Tyneside
(l1) Sunderland
Absolute difference ($|R_t^{(day)} - R_t^{(day-night)}|$)
0.00 0.25 0.50 0.75 1.00
Mar Apr May Jun Jul Aug Sep Oct Nov Dec
Mean
Median

Fig R3: Effect of timescale on the estimated connected R_t over the LTLAs of North East region. We consider two timescales: "day" (same as connected R_t as in Fig.5 in the main text), which are represented by the green curves in R3(a)-(l), "day-night", where unit time is half of a day and represented by the orange curves, and disconnected R_ts are shown by the red curves. In the "day-night" case, the first half of a day uses the mobility matrix as shown in SI Fig B(b), and for the second half we consider the identity matrix to account for the fact that people return to their home region at night. We quantify the difference between these curves of connected R_ts by calculating absolute residuals $|R_{t,i}^{(day)} - R_{t,i}^{(day-night)}|$ between green and orange curves (see Figs. R3(a1)-(l1)). Here, blue dots and purple crosses are the mean and median of the residual distribution at each time point, respectively. The corresponding error bars are represented in light blue color. We can see the choice of timescale does not lead to any fundamental change in the obtained results based on mobility flow across the regions.

We also referred to this in the main text of the revised manuscript **on page 13, lines 364-365** as follows:

Additionally, in SI Fig. M and N, we present an analysis showing that the main findings are robust to the choice of time scale.

Please, find below more detailed comments:

2. Abstract: “This analysis also investigates the spatial scalability of the framework, indicating that lower spatial resolution can diminish the effect of inter-regional mobility on the disease transmission, and we conclude that utilizing a finer spatial scale is advantageous on the basis of data availability and computational resources to obtain a better picture of the detailed transmission dynamics.” - Isn’t this the usual result? Is it possible to quantify somehow “how much better / worse” results are with a certain increase / decrease of resolution? Or explain it in terms of the significance of the mobility network?

Response: Thank you so much for this comment. The transmission dynamics in any region depends jointly on mobility flows and the epidemic situation of the connected regions. Consequently, the appropriate resolution is the scale at which these two components interact. When neighbouring subzones have similar epidemic conditions, mobility does not have such influence on the transmission dynamics, in contrast, when epidemic conditions differ, even small mobile populations can significantly affect

transmission dynamics. For illustration, we describe two scenarios: (i) **mobility with similar epidemic conditions** and (ii) **mobility with heterogeneous epidemic conditions** across subzones. These results show how mobility and epidemic situations of the regions jointly shape the transmission dynamics over spatial scale.

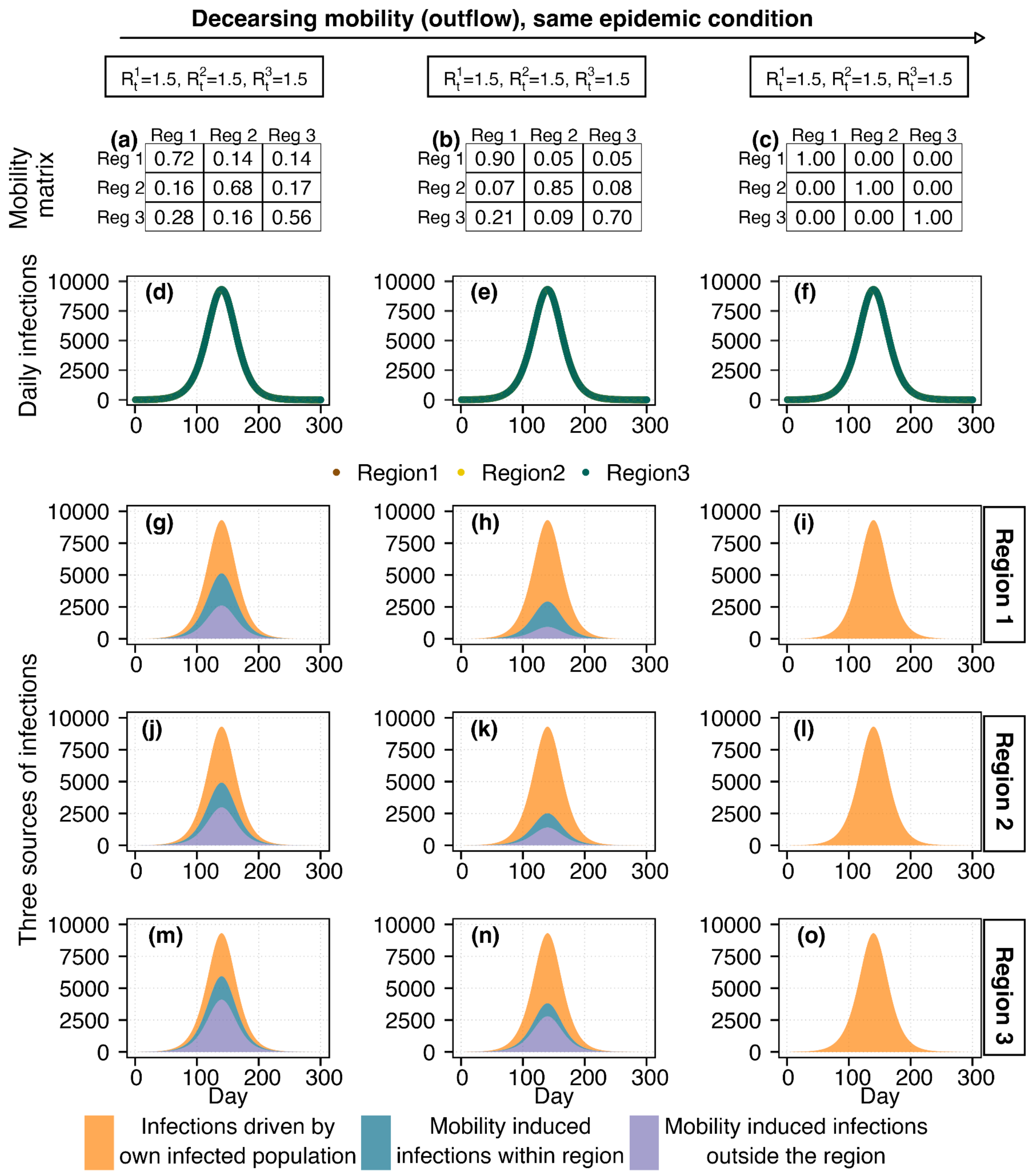

R4: Impact of mobility in a three-region system with identical epidemic conditions. Each region has an equal population of 1,000,000, the mobility matrices and R_t values are specified in the figure. From left to right, we consider the scenarios with decreasing outgoing mobility and increasing non-commuting population. In the rightmost panel (c) the regions are disconnected (no inter-regional travel), so the

mobility matrix is an identity matrix (all off-diagonal elements are zero). Figs. (d)-(f) show daily infections by region. Since the epidemic conditions are homogeneous, the incidence trajectories are identical across regions even when outflows differ. However, mobility redistributes the location of infection acquisition. Figs. (g)-(o) show that, although daily case counts are equal for the three regions, infections occur in different destination regions depending on the mobility pattern.

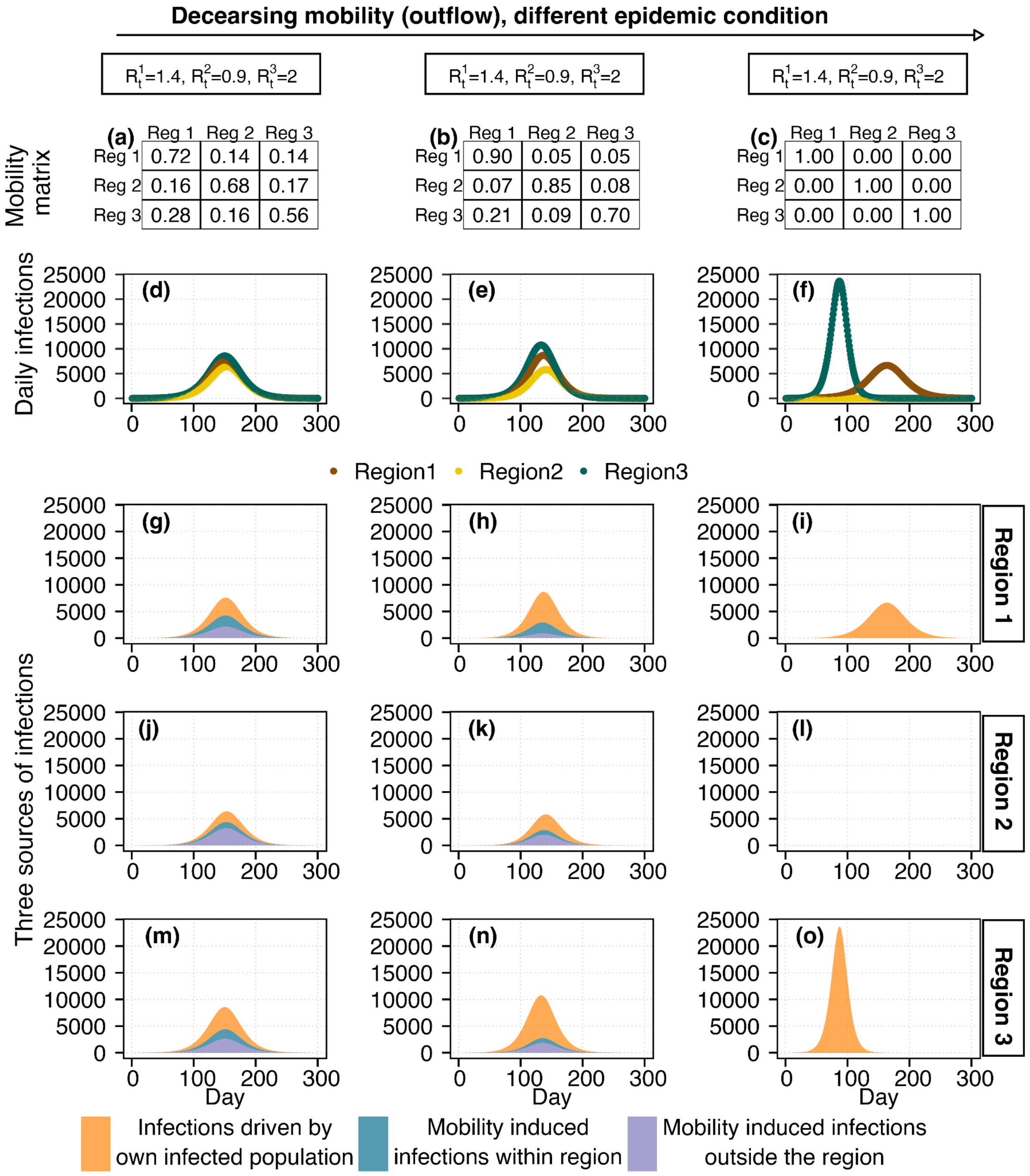

R5: Impact of mobility in a three-region system with heterogeneous epidemic conditions. Each region has an equal population of 1,000,000, the mobility matrices and R_t values are specified in the figure. From left to right, we consider the scenarios with decreasing outgoing mobility and increasing non-commuting population. In the rightmost panel (c) the regions are disconnected (no inter-regional travel), so the mobility matrix is an identity matrix (all off-diagonal elements are zero). Figs. (d)-(f) show daily infections by region. As the epidemic conditions are heterogeneous over regions, the incidence trajectories are different based on the mobility and local epidemic condition. For example, region 2 is with a controlled situation with $R_t < 1$, however, in Figs.(d) and (e), there is an outbreak because of the mobility-driven importation, and in the absence of any mobile population it fails to generate any outbreak (See Fig.(f)). Figs. (g)-(o) show the different sources of infections occur in different destination regions depending on the mobility pattern.

These simulations underscore that both human mobility and regional epidemic status jointly shape disease transmission dynamics. Mobility has a significant impact in heterogeneous epidemic scenarios, where differences in transmission intensity across regions can lead to outbreaks in low-risk areas through population movement. This is particularly relevant in real-world settings, where such heterogeneity is common during the early stages of an epidemic. As the epidemic becomes widespread and more uniform across regions, local disparities can appear due to the differences in policy implementation, healthcare capacity, demographic characteristics, or socio-economic conditions.

We added the above discussion in the **SI in Figs. A and B (pages 1-4)** and mentioned in the main text on **page 11, lines 313-314.**

We also modified the **lines in the abstract regarding spatial scales** in terms of mobility as follows:

We primarily validate the approach using simulated data, and demonstrate the limitations of estimating R_t without considering mobility. We then apply it to two real-world mobility settings using SARS-CoV-2 mortality data: the regions of England and the LTLAs of North East region of England, and uncover the mobility driven effect on transmission at different spatial resolutions.

3. Introduction, line 6: SARS-CoV-2.
4. Line 7: NPI, to be mentioned first the meaning of the acronym: “non-pharmaceutical interventions (NPIs)”.

Response: Thank you for the suggestions. We have modified the revised manuscript (**page 2, line 9-10**) accordingly:

during COVID-19 pandemic, lockdowns and other non-pharmaceutical interventions (NPIs) led to substantial reductions in mobility within any country, and accordingly disease transmission.

- Line 18: models… lead…

Response: Thank you for pointing it out. We have changed it in the revised manuscript accordingly (**page 2, line 21**).

5. Consider adding parenthesis in equation 2 for easier readability.

Response: We included parenthesis in equation 2 for better readability (**page 5**).

6. I assumed the “time” in the model is “daily” (discrete nature of the sum), but that could be a bit unrealistic w.r.t. the mobility patterns. I understand that each time step could be anything, in theory? Could it be specified? Human mobility does not usually affect the whole day.

Response: Thank you so much for the suggestion. We already address this point in the first comment. We have mentioned this in the context of the first comment in the main text on **page 5, lines 145-149** as follows:

In the context of the framework's timescale, if individuals do not spend an entire day in another region, the framework is flexible enough to adopt that fact. A detailed simulation is provided in Fig L in SI. However, in this work, we consider a daily time scale throughout the study, based on the available data.

7. A bit of repetitions in the text of the methods section: e.g. lines 184-185 explaining the meaning of the diagonal elements of the “m” matrix (people who stay in their region) was already implied before (line 143). Repetition again in lines 206-207.

Response: Thank you so much for the suggestion. We have removed the lines in the revised manuscript. To maintain an easier flow, we have instead added this information earlier **on page 4, lines 111-112**.

8. Personally, section 2.5 is not fully clear to me. You simulated Rt and not the cases in this experiment? And then the cases are generated from the mobility-inclusive renewal equation. Perhaps an extra line in the prior section specifying Rt will be simulated (assumed), and an extra line in this section explaining the choice to simulate Rt would improve the readability and clarity of the work.

Response: Thank you so much for identifying the fact that section 2.5 needs more clarification for the reader. We break down the methodology :

Simulation (explained in Section 2.4):

- We did not simulate R_t. We randomly choose three different countries (IND, GRB, ITA) from the repository https://github.com/covid-19-Re/dailyRe-Data (mentioned in the reference 38), and take the time series of R_ts for a time period of 350 days (as shown in Fig.2(c) in the main text).
- These are the R_ts for three regions we use to generate the simulated data (Daily infection as shown in Fig 2(d) in the main text) with the renewal equation represented by Eq. 2.

Estimation (explained in Section 2.5):

- Now, to validate the framework in a controlled settings, we estimate R_ts using the mobility matrix (Fig 2(b) in the main text) and simulated daily infection data (shown in Fig 2(d) in the main text). For this, we modeled R_t as a geometric random walk $log(R_t) \sim N(log(R_{t-1}), \sigma)$.
- For the prior we choose $log(R_0) \sim N(-0.1, 0.5)$ and $\sigma \sim N^+(0, 0.5)$.

For better readability of the manuscript, we modified and explicitly specified the prior in Sec.2.5. of the revised manuscript as follows (on **page 7, Section 2.5**):

R_t is modeled as geometric random walk, where at any time t, $log(R_t)$ is updated according to a normal distribution with the mean $log(R_{t-1})$ and a standard deviation σ as follows:

$$log(R_t) \sim N(log(R_{t-1}, \sigma)$$

Here, R_0 is the reproduction number at $t = 0$ defined as the average number of secondary cases coming from an infected individual at the start of the outbreak. It is assigned a weak prior $log(R_o) \sim N(-0.1, 0.5)$ to consider a wide range of values. $\sigma \sim N^+(0, 0.05)$ follows a prior of truncated normal distribution (constrained to positive values).

9. I miss a short explanation of the choices made in section 2.6 (some distributions, mean choices, etc.). Can be in the Appendix and referenced here.

Response: Thank you for the comment. We follow previous COVID-19 literature for the generation-time distribution, onset-to-death delay distribution and prior specifications. We added all the references listed below:

Distributions	References
Generation time distribution	• Flaxman et al. Nature. 2020;584(7820):257–261.<br>• Unwin et al. Nature communications.2020;11(1):6189.<br>• Bi et al. The Lancet infectious diseases. 2020;20(8):911–919.
Onset-to-death distribution	• Flaxman et al. Nature. 2020;584(7820):257–261.<br>• Unwin et al. Nature communications. 2020;11(1):6189.<br>• Verity et al. The Lancet infectious diseases. 2020;20(6):669–677.
Dispersion parameter	• Unwin et al. Nature communications. 2020;11(1):6189.
Infection fatality ratio	• Flaxman et al. Nature. 2020;584(7820):257–261.

$R_{0,i}$	• Flaxman et al. Nature. 2020;584(7820):257–261.
Initial Infections	• Unwin et al. Nature communications. 2020;11(1):6189.

We also included all the references corresponding to each distribution and parameter choices in Section 2.6.

10. For example, line 254: “The 95% credible intervals representing the uncertainty, are shown as the shaded bands…” – shouldn’t all these kind of explanations be restricted to the caption of the figure?

Response: Thank you for your suggestion. We have moved all of these descriptions to the captions of Fig. 3, 4, 5, 6, 7.

11. Results in Figure 3, aren’t they to be expected? The epidemic was simulated using the same equations that now predict Rt better (i.e. connected model). Perhaps results could be presented a bit differently: not in terms of “how much better this model fits w.r.t. to not considering mobility” (expected) but in terms of the risks of computing Rt without mobility and therefore misreading the real transmissibility of the epidemic – pointing out at specific scenarios when this happens (over/under estimation).
12. Sentence in rows 280-282 is a clear example of the point above.

Response: Thank you for the comment. We have modified the entire section and added two tables for additional quantitative support (as suggested by another reviewer) to the revised manuscript **(Section 3.1, pages 9-11)** as follows:

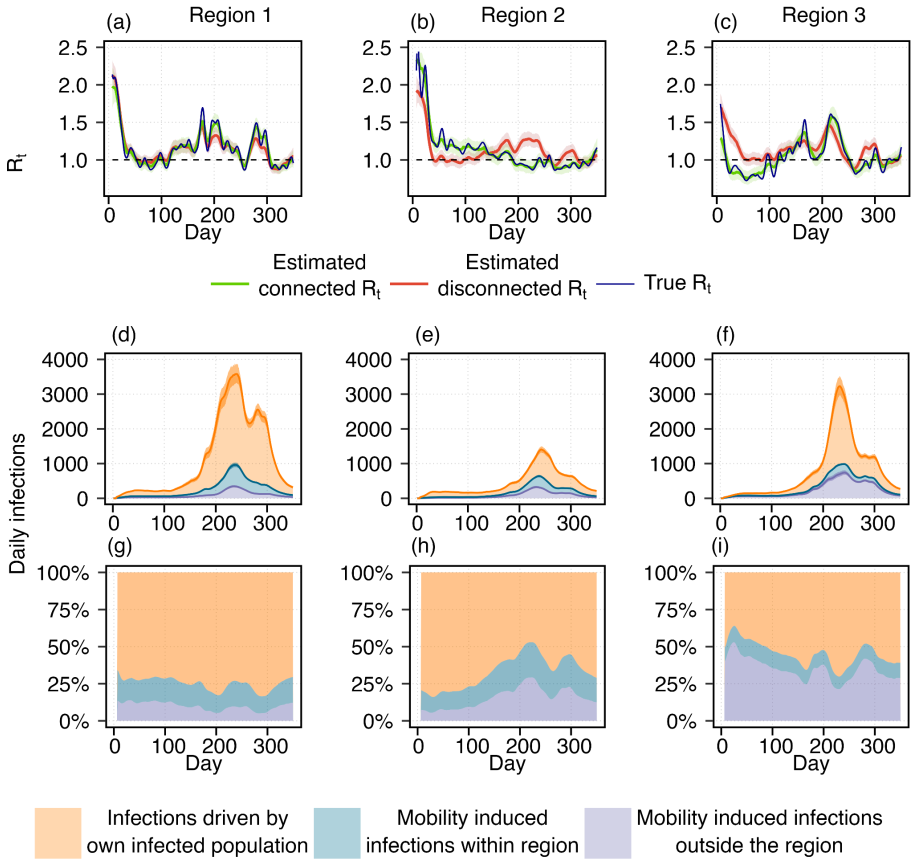

To validate the proposed framework, we compare the estimated R_ts with the ground truth used in the simulation. This validation ensures that the framework reliably captures the transmission dynamics under controlled conditions before applying it to real-world scenarios. Fig. (a)-(c) present the results for the simulated dataset, showing estimated reproduction numbers for three regions.

The disconnected and connected R_t estimates are shown in red and green, representing scenarios without and with inter-regional mobility, respectively. In the disconnected model, mobility-driven infections are ignored, assuming individuals remain within their respective regions and infections occur exclusively within regional boundaries. This simplification produces biased R_t estimates. Table 1 shows the correlation between one-step increments in R_t for the connected (green) and disconnected (red) estimates against the ground truth (blue). The consistently higher correlation between the connected R_t and ground truth supports the observation that

disconnected R_t can lead to a severe misleading understanding of transmission patterns.

Table 1. Correlation between one-step increments $(R_t - R_{t-1})$ of true and estimated R_ts.

		Connected Rt	Disconnected Rt
True Rt	Region 1	0.89	0.84
True Rt	Region 2	0.60	0.50
True Rt	Region 3	0.80	0.69

To further investigate the relationship between mobility-driven infections and overall transmission dynamics, in Fig. (d)-(i) show the absolute number and proportion of infections arising from three distinct sources. These figures indicate that a considerable proportion of infections is influenced by mobility. This effect becomes particularly important when R_t varies across regions and the epidemic behaves differently from one region to another, an effect overlooked by the disconnected R_t.

Table 2: Correlation between the residuals (disconnected R_t minus ground truth) and the proportion of mobility-driven infections.

		Residuals (Estimated disconnected R_t - Ground truth)
Region 1	Proportion of mobility driven infection	0.7284727
Region 2	Proportion of mobility driven infection	0.9428337
Region 3	Proportion of mobility driven infection	0.9385775

Table 2 produces a quantitative support that shows a positive correlation between the residuals of disconnected R_t with respect to the ground truth and the proportion of mobility-driven new infections. When the mobile population gets fewer infections in

other regions than they would have without commuting, disconnected R_t underestimates (residual negative), and if the number of such infections is higher, it overestimates the epidemic (residual positive). For example, around day 100, the disconnected R_t underestimates the true R_t in Region 2. Around that time, as 15% population of region 2 commutes to regions 1 and 3, where reproduction numbers are lower than region 2, it reduces mobility driven infection outside the region, and as well as the total number of infections in region 2. However, disconnected R_t fails to capture this mobility-driven lowering effect outside the region and leads to an underestimation of the true epidemic situation. On the other hand, around day = 220 in region 2, by failing to account for that mobility-driven increasing infection outside the region, disconnected R_t overestimates the epidemic situation. Therefore, disconnected R_t underestimates or overestimates an epidemic when mobility reduces or increases infections compared to a no-mobility scenario. Further discussion of the effects of mobility and regional epidemic conditions on transmission dynamics is provided in Figs A and B of SI.

13. Lines 288-296: again, shouldn't it be just in the caption of the figure? + Some of those panels described in these lines were already mentioned before. As a reader, I already went through those graphics and now it feels like an unnecessary / misplaced / repetitive explanation.

Response: Thank you for your suggestion. We have moved all of these descriptions to the caption of the figures.

14. Section 3.2: slightly repetitive / long to present a single and clear result: no significant differences observed when mobility affects only a small % of the studied population.

Response: Thank you for the suggestion. We have moved the description of the figure to the caption. We also added a quantification (absolute difference between connected and disconnected R_t, as suggested by another reviewer) to the **SI in fig. H** and mentioned in the main text in **Sec. 3.2 on page 11** in the revised manuscript to make it more concise as follows:

We observe that the connected and disconnected estimated Rts are nearly identical across all regions. The absolute difference between connected and disconnected R_ts are shown in SI Text in Fig H. After an early discrepancy for a few regions (East,

London, South-east) maximum mean absolute difference reaches 0.25. This suggests that at regional scale, mobility does not have a significant impact on infections after the early transmission phase. Upon examining the mobility matrix (see Fig C(a) in SI Text), it is observed that the fraction of the population commuting between regions is very small relative to the total population of each region. This makes the regions of England treatable as disjoint areas, where the disconnected model is sufficient for Rt estimation at this spatial scale. Transmission at the regional level can be treated as homogeneous spreading within regions, with minimal influence from inter-regional mobility. This suggests that, when the disease is already established in all regions, mobility can be reasonably excluded from models at such spatial scales.

15. Section 3.3: Personally, I see this section as one of the key results of this work. Perhaps I missed it, but is the mobility matrix different during lockdown periods? I believe it should change when lockdown was in place. From Figures 6 and 7, it seems mobility was not considered (mij = 0 for i!=j) during lockdown? Please specify it in the text how were lockdowns dealt with (full lockdown, reduced mobility, …).

Response: Thank you so much for the comment. We agree that it was not mentioned earlier. We have now addressed this by adding the following line on **page 8, lines 221-223** of the revised manuscript:

During the lockdown periods, we set the mobility matrix to identity ($m_{ij} = 1$ if $i = j$, and 0 otherwise), ignoring inter-regional mobility.

16. Lines 352-355: in the caption.

Response: We have moved this to caption in the revised manuscript.

17. The discussion in terms of “finer resolutions” (line 369, for example) shouldn’t it be more accurate saying: when > xx% of the population commutes between studied regions. I understand getting a specific percentage as a threshold might not be possible / easy, but perhaps the text would benefit from a discussion in terms of “presence / absence of significant mobility” and not only in terms of “resolution”. It has been better described in the discussion section already. Also, because, if the “scale is too fine”, the underlying assumption of homogenous mixing inside each region would become problematic. Perhaps add this as a limitation in the discussion.

Response: We really appreciate the comment. We have removed this line in the revised manuscript, and we discussed the spatial resolution for England in terms of a substantial inter-regional mobile population. For example, in discussion we modified the line regarding this (on **page 16, lines 405-408**) as follows:

The situation changed markedly at a different spatial resolution. Focusing on the 12 Lower Tier Local Authorities (LTLAs) within the North East region, we observed substantial inter-LTLA mobile population and, correspondingly, a clear difference between connected and disconnected Rt estimates.

We also added the limitation to the discussion in the revised manuscript (on **page 17, lines 448-453**) as follows:

Specifying a particular mobility threshold that impacts disease transmission is beyond the scope of this work, as we obtained that transmission dynamics are jointly shaped by the mobility and epidemic situations across different regions. In addition, the choice of spatial resolution requires careful attention, as at very fine spatial scales, the assumption of homogeneous mixing within each region may be violated.

18. Slight "double sense" that came to my mind: finer scale in terms of more regions or in terms of smaller regions? I bet at a scale in which mobility is significant. Just try to be clear on that.

Response: Thank you for highlighting this. We sincerely checked the manuscript and clarified this point throughout. We have removed the term "finer" when referring to spatial scale, and changed the wording related to spatial resolution. We also discuss this point elaborately in our response to comment no 2.

19. Lines 452-453: "within" alone (no "into"). // In regions with commuting populations, …

Response: Thank you so much for identifying it. We have changed this in the revised manuscript (**on page 17, line 455**).

20. Lines 461-463: add as limitation in the discussion.

Response: Thank you for the suggestion. We have removed this part from the manuscript as we found that it refers to a future work on computational aspects without showing any baseline in this work (as also suggested by another reviewer).

Reviewer #2:

Review of “Incorporating human mobility to enhance epidemic response and estimate real-time reproduction numbers” by Roy et al.
This article proposes an extension of the renewal equation approach for Rt estimation that includes the effect of commuting mobility. The proposed framework is first validated in a controlled environment using synthetic data, and then applied to a real-world case study considering reported death data from 2020 COVID-19 waves in England.
Overall, my review for this article is positive. Reliable Rt estimates are of clear importance especially when facing emerging outbreak such as COVID-19, and tools that allow corrections for the impact of human mobility are important and needed. However, in the current status the paper is missing key information on the analysis and proper quantitative justifications of the main claims. These issues can be nonetheless approached with a review of the manuscript as they do not constitute fundamental flaws in the proposed approach to my judgement.
Please find below my comments aimed at improving the presentation and the justification of the findings.

Response: We really appreciate your positive feedback, and we are glad that you found our work valuable in the context of an emerging epidemic. We carefully followed all the suggestions and incorporated them into the revised manuscript. A detailed discussion is provided below:

Introduction
1. The justification for the need for the proposed approach appears weak. The message that emerges is that mobility is important when modeling the transmission of infectious diseases, but no further explanation and/or examples is provided. Further details on how mobility (especially commuting, which is the type of mobility considered in the paper) can affect the spread (and as a result the estimation of Rt) would significantly strengthen the support for the proposed approach. My feeling is that the current justification for the need of the inclusion of mobility in the renewal equation approach is too shallow and with little effort it can be given more depth.

Response: Thank you so much for the suggestion. Regarding this comment, we have included a part to the introduction section to the revised manuscript on **page 2, lines 4-14** as follows:

While long-range mobility like air-traffic flow between countries quantifies the importation risks for a country, daily commuting travel structure builds the onward transmission routes within a country after initial importation [6-8]. By daily commuting flows, people move across different regions and increase contact opportunities at distant places, that enables inter-regional disease spread. In contrast, during COVID-19 pandemic, lockdowns and other non-pharmaceutical interventions (NPIs) led to substantial reductions in mobility within any country, and accordingly disease transmission [9-10]. This highlights the need to account for mobility in the real-time estimation of reproduction numbers, and therefore, ignoring mobility in transmission models can lead to biased parameter estimates and compromise the accuracy of future predictions.

2. The comparison with other modeling approaches lacks a fundamental class: metapopulation models. Metapopulation models explicitly includes a spatial component as well as mobility, therefore it seems natural to me to mention them, with strengths, limitations, and comparison with the renewal equation approach

Response: Thank you for the suggestion. We have mentioned it on **page 2, lines 26-31** in the revised manuscript. We modified the lines as follows:

In this context, metapopulation model partitions the total population into subpopulations, assuming homogeneous mixing within each subpopulation and mobility between them [4,16]. Although several challenges [17] have been associated with this approach, it is widely used to study the mechanism behind an outbreak, rather than to evaluate the real time transmission dynamics.

3. An entire paragraph is dedicated to the discussion of the availability of mobility data as well as models of human mobility. This seems misplaced to me. Indeed, the main contribution of this paper is methodological, therefore I do not see the need to include these practical considerations in the introduction. They may be instead relevant for the discussion and the conclusion, when authors discuss further application and expansion of their framework.

Response: Thank you so much for pointing this out. We removed the paragraph and added one line related to it in the introduction **on page 2, lines 33-35** in the revised manuscript as follows:

In practice, studies often use proxy-mobility datasets, such as, mobile-phone, public-transport records [20-21], or substitute established mobility models when empirical data are unavailable [22-24].

We also mentioned in the discussion **on page 16-17, lines 441-448** in the revised manuscript as follows,

A significant challenge when working with mobility-induced transmission models is the limited availability of mobility data. Various datasets serve as proxy for human mobility with its advantages and limitations, at this stage in our case study, we relied on publicly available data to mitigate data constraints. However, these data are often aggregated and may not reflect real-time changes in movement patterns, such as those induced by behavioral shifts or NPIs. Future work could enhance this model by integrating dynamic mobility data, such as mobile phone-based metrics, and extending it to capture other heterogeneities, such as age structure or vaccination coverage.

Materials and Methods

4. I think it would be more correct to refer to g as the generation time distribution (also throughout the text)

Response: Thank you for the suggestion. We also agreed that "generation time distribution" would be more correct. We have changed g as generation time distribution throughout the revised manuscript.

5. The integral definition of gs needs some more explanation for the extremes of the integral

Response: Thank you for the comment. Regarding the integral definition of g_s, we discretize the continuous density function $g(t)\,(t > 0)$ into daily probabilities $g_s = \int_{s-0.5}^{s+0.5} g(t)dt,\ s = 2, 3, 4,$... and $g_1 = \int_{0}^{1.5} g(t)dt,$

Where, $g(t) \sim Gamma(6.5, 0.62)$. The first bin is left-truncated at 0, while subsequent bins have width 1 and are centered at the corresponding integer days with $\sum_s g_s = 1$.

6. Line 110, when introducing the mobility matrix m, I suggest also specifying how to interpret the diagonal element (i.e., the fraction of non-commuting individuals), otherwise some readers may assume that mii = 0 only to find out later that this is not the case.

Response: Thank you for the comment. We have added one line as follows on **page 4, lines 111-112** in the revised manuscript:

The diagonal elements of the mobility matrix m_{ii} indicate the fraction of the population that does not commute, staying within their respective regions.

7. In the simulated data generation section, it is not clear which values of Rt have been used and how they have been chosen

Response: Thank you for identifying this point. We randomly choose three different countries (IND, GRB, ITA) from the repository https://github.com/covid-19-Re/dailyRe-Data (mentioned in the reference 38), and take the time series of R_ts for a time period of 350 days (as shown in Fig.2(c) in the main text). We also mentioned this to the revised manuscript **on page 6, lines 189-191**.

8. In line 203 R0 is introduced for the first time. I understand that this is R(t=0), but given the epidemiological importance of this quantity I suggest to spend a couple of words to explicitly say what R0 is in this context

Response: Thank you for the suggestion. We added this to the revised manuscript (**page 7, line 206**) as follows:

R_0 refers to the reproduction number at $t = 0$ defined as the average number of secondary cases coming from an infected individual at the start of the outbreak.

9. Given how central mobility is, I suggest providing more details on the mobility matrices used in the England case study (from where they are taken, how data is collected, which year, etc). Lines 215-217

Response: Thank you for your suggestion.

- The mobility data we obtain from https://www.nomisweb.co.uk/census/2011/wf02ew, which provides 2011 census estimates of the residents of England and Wales aged 16 and over in employment the week before the census. It represents the location of the usual residence and place of work.
- We use the data accordingly for a considered spatial scale (regions or LTLAs) and calculate the proportions of outflows in order to obtain the mobility matrix compatible with our model.

We also described and added the link as a reference in the revised manuscript (**page 8, lines 217-221**) as follows:

The mobility matrix (see Fig C(a) in SI) is constructed over the nine regions using data from [40], which reports 2011 census estimates for individuals aged 16 years and over in England and Wales with their place of residence and workplace. We calculate the proportions of outflows to obtain the mobility matrix that is compatible with the model 2.

The dataset is additionally cited on page 9 in the context of the mobility matrix over the LTLAs of north-east region.

10. The use of mobility data from the Google mobility report to modulate transmission should be justified and supported with references, see for example: https://www.pnas.org/doi/10.1073/pnas.2421993122, https://www.nature.com/articles/s42254-021-00407-1

Response: Thank you for the suggestion. We modified the main text (**page 8, lines 238-257**) in the following way:

To estimate the instantaneous reproduction number $R_{t,i}$ for each region, we account for temporal changes in human behavior utilizing activity trends derived from the google mobility report [9]. We use five different types of information from Google mobility data: retail and recreation, groceries and pharmacies, parks, transit stations, and workplaces. Here, we define R_t as follows:

$$R_{t,i} = R_{0,i}.f(-X_i P_t \alpha_{t,i} - \epsilon_{w_{t,i}}),$$

where, $f(x) = 2exp(x)/(1 + exp(x))$, is twice the inverse logit function. $R_{0,i} \sim N(3.28, \kappa)$ with $\kappa \sim N^+(0, 0.5)$ for all regions. Here, $R_{0,i}$ is the initial reproduction number for region i. In this context, it is the value of the reproduction number before fitting starts. $\epsilon_{w_{t,i}}$ is the weekly effect that follows a random walk centered at 0, with the week index $w_{t,i} = [(t - t_0)/7] + 1$, where t_0 is the first day of seeding. X_i is the region-specific effect for covariates $\alpha_{t,i}$, which are the averages of five different dimensions observed from Google mobility data. The implementation of NPIs typically leads to a significant decline in both population mobility and disease transmissibility. However, in many countries, the adoption of social distancing behaviours weakened the correlation between mobility and transmissibility following the relaxation of control measures [44,45,46]. To account for this effect, we introduce the parameter P_t, which captures behavioural changes associated with different lockdown phases, with distinct values x, y, and z representing the periods up to the end of the first lockdown, from the end of the first lockdown to the end of the second lockdown, and the post-second lockdown period, respectively.

We also added the reference https://www.nature.com/articles/s42254-021-00407-1 and https://www.pnas.org/doi/10.1073/pnas.2421993122 **on page 8, line 253**.

11. The functional form of Rt (~line 226) is never justified. What is the rationale behind this choice? Is this a standard approach (if so, relevant references should be added)? Did you perform any sensitivity analysis to assess the impact of this choice?

Response: Thank you so much for the comment. The similar functional form of R_t were also considered in the previous literature, such as:

1. **Flaxman S, Mishra S, Gandy A, Unwin HJT, Mellan TA, Coupland H, et al. Estimating the effects of non-pharmaceutical interventions on COVID-19 in Europe. Nature. 2020;584(7820):257–261.**
2. **Unwin HJT, Mishra S, Bradley VC, Gandy A, Mellan TA, Coupland H, et al. State-level tracking of COVID-19 in the United States. Nature communications. 2020;11(1):6189.**

We modified the model according to our study and the available data. The explanation behind this functional form of R_t is explained in the answer of the last question. However, a small and concise description is as follows:

$R_{t,i}$ is modeled as $R_{t,i} = R_{0,i} f(-X_i P_t α_{t,i} - ε_{w_{t,i}})$

Here, $R_{0,i}$ denotes the reproduction number at the start of the fitting period. We set $f(x) = 2exp(x)/(1 + exp(x))$, i.e., twice the inverse-logit function. The inverse-logit ensures $R_{t,i} > 0$ and the factor of 2 ensures that, when there is no effect from Google mobility or the weekly term, $R_{t,i} = R_{0,i}$.

All the details and the supporting literature are mentioned in the main text **(page 8, lines 238-257)** in the revised manuscript.

12. Details on model fitting procedure are completely missing. Relevant information on model calibration should be added in both the main text and if needed in the Supplementary Information

Response: Thank you for this comment. For the model fitting, the weekly death data $D_{w,i}$ for region i in week w is modeled using a negative binomial distribution:

$$D_{w,i} \sim Negative\ binomial(d_{w,i}, d_{w,i} + d^2_{w,i} ψ)$$

Here, $ψ \sim N^+(0, 5)$. Here, $d_{w,i}$ is the expected number of weekly deaths, calculated as the sum of daily deaths over seven days.

This is described on **page 9, lines 231-234**.
For this, we use CmdStanR with NUTS sampler. 4 chains are generated in parallel for 500 iterations after 1000 warm-up. There are no divergent transitions. We mentioned this on **page 9, lines 274-275**. We also added one figure with the distribution of Rhat **in SI Fig O** showing that Rhat < 1.01.

Results
Overall, the comparisons between estimated Rt are at the moment purely visual, qualitative, and anecdotal. I believe that with relatively low efforts the comparisons can be also made in quantitative terms and as result solidify the narrative

13. Figure 3a/b/c. The deviation between the red and the green curves can be better characterized, at the moment it is purely visual and not quantitative. A possible solution would be to compute correlations between one-step increments in Rt (i.e., (Rt) - (Rt-1)) of green and blue curves (e.g., ground truth vs connected Rt) and of blue and red curves (e.g., ground truth vs disconnected Rt). I expect higher correlations between one-step increments of green and blue curves, and this would further support the claim that the connected Rt provides a better estimate of the ground truth.

Response: Thank you so much for your suggestion to provide a quantification for the results represented in Figure 3(a),(b),(c) in the main text. We have added it in the revised manuscript on **page 9, lines 287-291** as follows:

Table 1. Correlation between one-step increments ($R_t - R_{t-1}$) of true and estimated R_ts.

		Connected Rt	Disconnected Rt
True Rt	Region 1	0.89	0.84
True Rt	Region 2	0.60	0.50
True Rt	Region 3	0.80	0.69

Table1 shows the correlation between one-step increments in R_t for the connected (green) and disconnected (red) estimates against the ground truth (blue). The consistently higher correlation between the connected R_t and ground truth supports the observation that disconnected R_t can lead to a severe misleading understanding of transmission patterns.

14. Similarly, in lines 288-314 there is a long discussion on the impact on mobility which is only supported by visual/anecdotal evidence. A possibility to make the discussion more quantitative can be the following. You can correlate, in each region, the (absolute) residuals of the red curve with respect to the ground truth (i.e., the error made by the disconnected model) with the percentage of new infections induced by mobility for that region (i.e., the information in panels 3g,h,i). I expect positive correlations, suggesting that the disconnected model makes most of the errors when the contribution to infections due to mobility is higher.

Response: Thank you so much for the suggestion. For a quantitative support of the result, we calculated the correlation between the difference in red curve (estimated disconnected R_t) and the blue curve (ground truth) with the proportion of mobility driven infection at that region. Please note that the residuals are signed differences, not absolute values. We have added this in the main text on **page 11, lines 298-314** as follows:

Table 2: Correlation between the residuals (disconnected R_t minus ground truth) and the proportion of mobility-driven infections.

		Residuals (Estimated disconnected R_t - Ground truth)
Region 1	Proportion of mobility driven infection	0.7284727
Region 2	Proportion of mobility driven infection	0.9428337
Region 3	Proportion of mobility driven infection	0.9385775

Table 2 produces a quantitative support of the above discussion that shows a positive correlation between the residuals of disconnected R_t with respect to the ground truth and the proportion of mobility-driven new infections. When the mobile population gets infection in other regions less than it would have if they do not commute, disconnected R_t underestimates (residual negative) the epidemic, and when they are higher, it overestimates (residual positive). For example, around day 100, the disconnected R_t underestimates the true R_t in Region 2. As the 15% population of region 2 commutes to regions 1 and 3, where reproduction numbers are lower, it reduces mobility driven infection outside the region, and as well as the total number of infections in region 2. However, disconnected R_t fails to capture this mobility-driven lowering effect outside the region and leads to an underestimation of the true epidemic situation. On the other hand, around day = 220 in region 2, the commuting population acquires more infections outside the region than they would have within region 2. That increases the mobility-driven infections outside the region, in turn, the overall infections in region 2. Again, by failing to account for that mobility-driven increasing effect, disconnected R_t

overestimates the epidemic situation at region 2. Therefore, disconnected R_t underestimates or overestimates an epidemic when mobility reduces or increases infections compared to a no-mobility scenario.

15. Figure4 and Figure5. It would be beneficial to show (either within the plot or in the SI) the (absolute) residuals between green and red curves, in order to be able to actually quantify the difference between the two estimated Rts. Mean, medians, and other statistics of these residuals can be mentioned when authors comment on the small difference between the curves.

Response: Thank you so much for the suggestion. We have added two figures **in SI in figs H and K** with the absolute residuals between connected and disconnected estimated R_ts of England and North East region of England as follows:

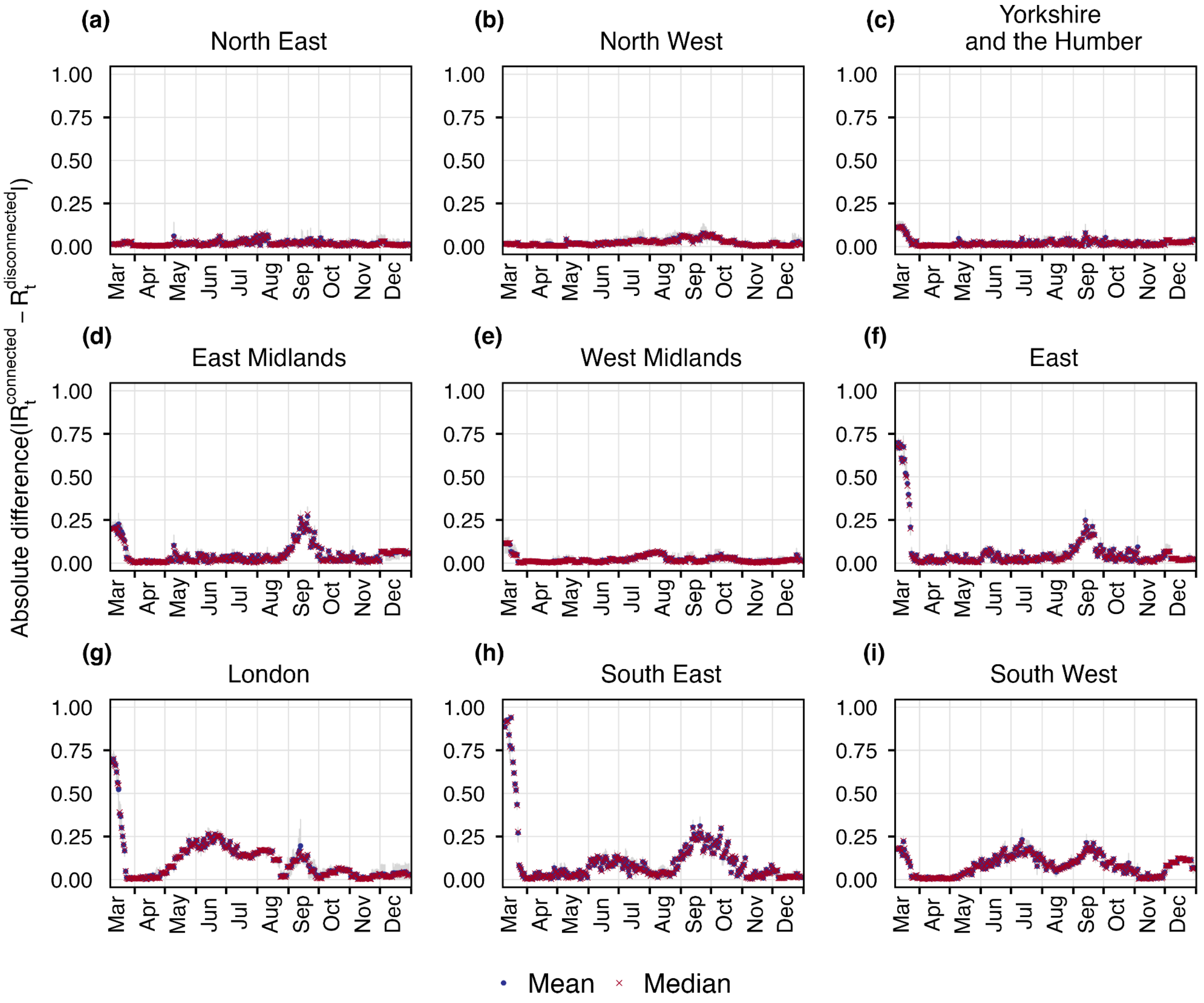

Fig R4: **Absolute residuals between connected and disconnected R_ts (shown in Fig.4 in the main text) for 9 regions of England.** Blue dots and red crosses show the mean and median of the absolute differences at each time point. Grey bars show the corresponding 95% credible intervals. Since the posteriors for the connected and disconnected R_ts are estimated independently, we first sorted each set of posterior draws in ascending order and then computed their element-wise difference.

We also mentioned this **on page 11, lines 326-330** in the revised manuscript as follows:

The absolute difference between connected and disconnected R_ts are shown in SI in Fig G. After an early discrepancy for a few regions (East, London, South East) maximum mean absolute difference reaches 0.25. This suggests that mobility does not have a significant impact on infections at the regional scale.

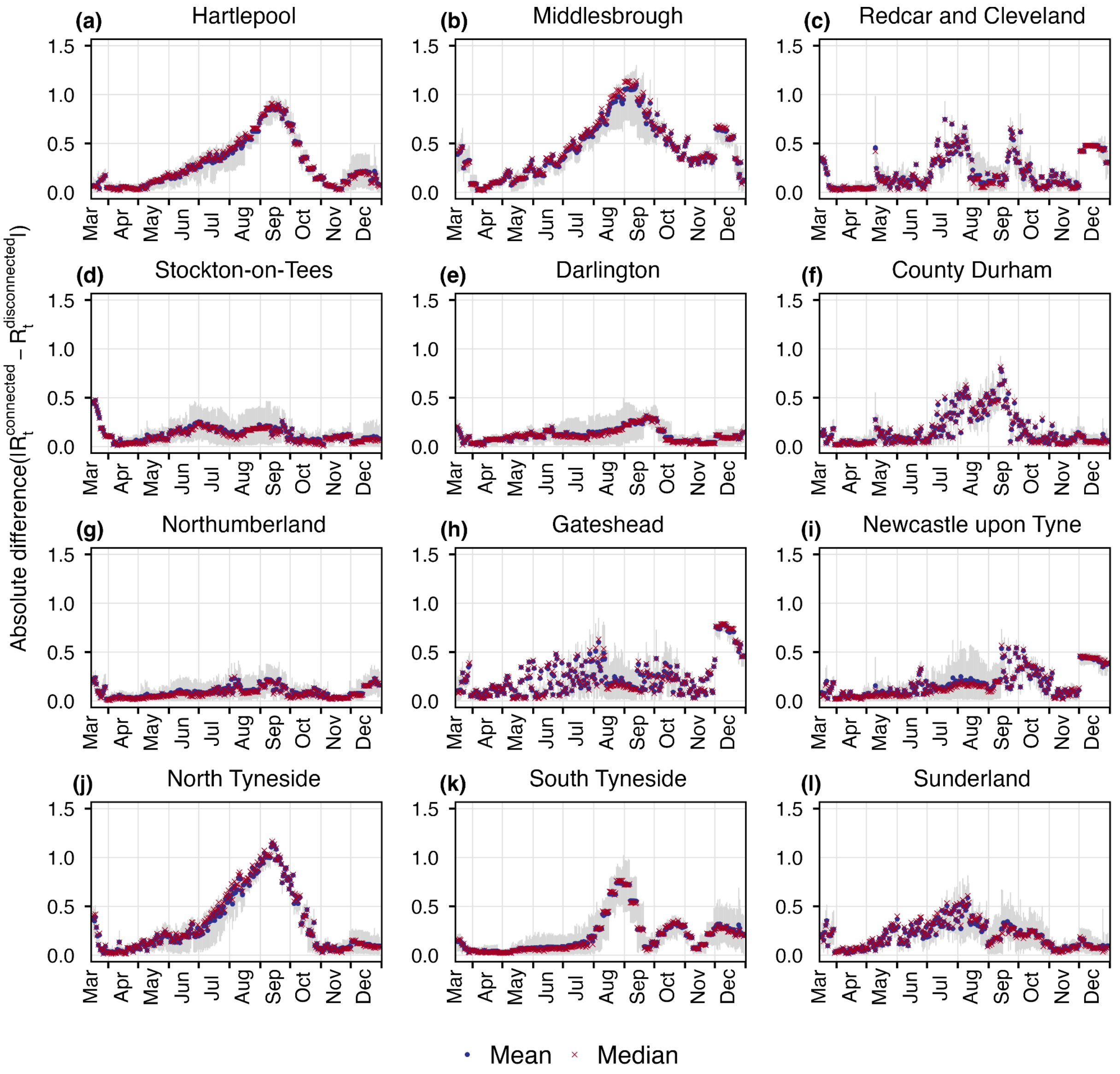

Fig R5: **Absolute residuals between connected and disconnected R_ts (shown in Fig.5 in the main text) for 12 LTLAs of the North East region of England.** Blue dots and red crosses show the mean and median of the absolute differences at each time point. Grey bars show the corresponding 95% uncertainty intervals. Since the posteriors for the connected and disconnected R_ts are estimated independently, we first sorted each set of posterior draws in ascending order and then computed their element-wise difference.

We also mentioned this on **page 12, lines 345-348** in the revised manuscript as follows:

Significant differences in the connected and disconnected reproduction numbers are observed in LTLAs such as Hartlepool, Middlesbrough, and North and South Tyneside. The absolute difference between connected and disconnected R_ts are shown in SI in Fig J. In Middlesbrough, the difference reaches 1.09 (0.7-1.3) in the second week of September. Similarly, for North Tyneside it reaches 1.15 (1.03-1.2).

Discussion/Conclusions

The paper concludes with a discussion on computational resources and further direction in this sense. I find this unnecessary, especially because information about computational intensiveness of the proposed approach is never discussed in the paper, without setting a baseline for future developments.

Response: Thank you so much for the suggestion. We agree on this point and have removed this part in the revised manuscript.

Other comments:

16. I apologise if I missed them, but references to the data used are completely missing, namely death data from England, population of the regions study, mobility matrices, citation to the Google mobility report data. All these references should be opportunely cited.

Response: Thank you for the suggestion. We have incorporated all the references of the data. In the revised manuscript, the corresponding reference numbers are as follows:

- Reproduction number for simulated data: [38]
- Death data from England: [42]
- Population of the regions: [41]
- Mobility matrices: [40]
- Google mobility report data: [9]

17. The manuscript would benefit from a thorough proof-read as there are some repetitions, typos, incorrections. Some examples - 55-59, these sentences repeat the same concept twice

- 159: should be “detailed”
- Equation 4 is missing top extreme of the summation in k
- Title of section 2.3, there’s a “:” in the title

- Line 334: I would suggest avoiding the term insignificant as no statistical test has been performed
- Line 374, “reductive factor” of what?
- Line 381, avoid using etc.
- Line 382, SARS-CoV-2 (- missing)

Response: Thank you for the suggestion. We have done a thorough check of the manuscript and modified it accordingly.
