# Supplementary material for: Incorporating human mobility to enhance epidemic response and estimate real-time reproduction numbers
Source: PLoS Comput Biol. 2025 Nov 13;21(11):e1013642. doi: 10.1371/journal.pcbi.1013642 (PMC12614531; doi:10.1371/journal.pcbi.1013642)
Supplement: Attachment — Submitted filename: Response to reviewer2.pdf [file pcbi.1013642.s018.pdf]

To
The Academic Editor and Section Editor,
PLOS Computational Biology

Thank you for your email of October 6, 2025, and for sharing the reviewers' feedback on our revised manuscript, "**Incorporating human mobility to enhance epidemic response and estimate real-time reproduction numbers" (PCOMPBIOL-D-25-00829R1)".**

We are grateful to the reviewers for their time, thoughtful comments, and kind acknowledgment that our revisions addressed all their concerns. We have incorporated every new suggestion carefully, and all changes are marked in blue in the revised manuscript and SI. For a detailed discussion on reviews, please refer to the Response to Referees. We show all the comments in **red,** responses in **black,** and all the changes to the revised manuscript and SI are highlighted in **blue**.

We truly appreciate the opportunity to improve the paper and hope the updated version is now suitable for publication.

Sincerely,
Mousumi Roy, on behalf of all co-authors

Response to Referees

We thank reviewers for their time and efforts. We appreciate their comments in improving our work. Below we show all the comments in **red,** responses in **black**, and all the changes to the revised manuscript and SI are highlighted in **blue**.

Reviewer #1:

Thank you for the detailed responses and the throughout revision of my comments. I'm in general satisfied with the changes made on response to my initial comments, and I would suggest it for publication.

Response: Thank you for your thoughtful review and for taking the time to evaluate our revisions. We are truly glad to hear you are satisfied with our responses and that you recommend the manuscript for publication.

As noted below, we have carefully addressed all your comments and incorporated the suggested changes into the revised manuscript.

Only a couple of additional comments:

Fig R3 shows in some cases non-negligible differences on the estimation of Rt. Although is true that, in general, Rt presents a similar trend by considering as time unit either 1 or ó a day, differences of up to ~0.7 were observed, which can lead to quite different transmission outcomes. Also, finding somewhat different Rt's when considering different mobility patterns seems reasonable, and somewhat the aim of your work. Perhaps it would be better to say that in the absence of mobility data at a finer scale than "daily", results seem still robust when comparing them to a potentially "more realistic scenario" (day-night).

Response: Thank you for this insightful comment. You are absolutely right that Fig. R3 (S13 Fig. and S14 Fig.) shows non-negligible differences—up to ~0.7 in some cases—between the R_t estimates obtained using "day" versus "day-night" time scales. We agree that such differences could imply distinct transmission interpretations in specific contexts**.**

That said, our core finding remains robust: even in the absence of sub-daily mobility data, the daily-scale R_t estimates capture the overall epidemic trajectory consistently with the more granular "day-night" scenario. As you suggest, this supports the practical utility of daily mobility data when finer-resolution data are unavailable.

In response, as per your suggestion we have revised the caption of S14 Fig.(page no 20, line no 593-595) to clarify this point:

Therefore, in general results obtained using daily time scale seem robust when comparing them to a potentially more realistic time scale (day-night).

We appreciate your suggestion, it helped us strengthen the interpretation of this comparison.

Perhaps I am missing something, but section 2.5 is still not clear to me. How are you exactly estimating Rt? Via an MCMC or ABC or…? I miss it to be clearer in this section how the fitting is done, other than the priors and "jumps". From what I read in page 9,

you are using the software CmdStan for model fitting? Try to be organized and clear on the methodological details.

Response: Thank you for this helpful comment. We agree that Section 2.5 needed greater clarity on the estimation procedure. We have revised it to explicitly describe how R_t is inferred.

In brief, we estimate R_t using a Bayesian hierarchical model implemented in CmdStan, with the No-U-Turn Sampler (NUTS), an adaptive Hamiltonian Monte Carlo algorithm. The model links observed case incidence to time-varying reproduction numbers through a renewal equation, with R_t modeled as a Random Walk process. Priors and hyperpriors are specified as outlined in the original text, and the posterior distribution of R_t is obtained via MCMC sampling.

In the updated Section 2.5 (page no 7, line no 210-212) we now state:

We use CmdStan [ref. 42 in the main text] for model fitting, with NUTS sampler. 4 chains are generated in parallel for 500 iterations after 500 warm-up with no divergent transitions.

We hope this clarification makes the estimation workflow more transparent. Thank you again for pointing this out.

Reviewer #2:

I would like to thank the authors for successfully addressing all of my previous points. Methods are now clearly explained and results are supported by quantitative evidence.

Response: Thank you for your kind and encouraging feedback. We're very glad to hear that our revisions have clarified the methods and strengthened the support for our results. We truly appreciate your time and thoughtful comments.

I have a couple of (very) minor suggestions on novel additions to the introduction:

1) Lines 20-22: in more advanced compartmental models we can relax, at least partially, the assumption of homogeneous mixing via strategies such as contact matrices. As such, I think it would be more correct to say "Compartmental mechanistic models [11], in the absence of a spatial component and more advanced mixing

assumptions (e.g., contact matrices) may lead to biased estimation due to their assumption of homogeneous mixing, a condition that rarely reflects the real-world mixing pattern."

Response: Thank you for this excellent suggestion. We agree that the original phrasing oversimplified the capabilities of modern compartmental models. We have updated lines 20-24 on page 2 to read:

Compartmental mechanistic models [ref. 11 in the main text], in the absence of a spatial component and more advanced mixing assumptions (e.g., contact matrices) may lead to biased estimation due to their assumption of homogeneous mixing, a condition that rarely reflects the real-world mixing pattern [refs. 12,13 in the main text].

We appreciate your careful reading and the opportunity to improve the accuracy of this statement.

2) Similarly for the metapopulation model. It is not necessary to assume homogeneous mixing within subpopulations. As such, that claim can be removed or relaxed. I also think the following should be either removed or changed "...approach, it is widely used to study the mechanism behind an outbreak, rather than to evaluate the real time transmission dynamics". There are notable examples of metapopulation approaches used to study real-time dynamics of infectious diseases, especially at the beginning of emerging outbreaks when it is crucial to estimate importation routes and probabilities, see for example: https://www.science.org/doi/10.1126/science.aba9757, https://pmc.ncbi.nlm.nih.gov/articles/PMC3585792/

Response: Thank you for this insightful comment and for pointing us to those relevant examples. You are absolutely right that metapopulation models do not inherently assume homogeneous mixing within subpopulations, and that they have been successfully used to study real-time transmission dynamics, particularly during emerging outbreaks.

In response, we have revised the text on page 2 (line 29) to remove the imprecise statement and better reflect the flexibility and real-time applicability of metapopulation approaches and cited the relevant references as well. We appreciate your careful reading and the opportunity to correct this oversight.

The added text on page 2 at line 27-33, now reads as:

Metapopulation model partitions the total population into subpopulations, with mobility between them [refs. 4, 16 in the main text], often used to understand the mechanism and study real-time transmission in a spatially explicit setting [refs. 17, 18 in the main text]. However it is associated with several challenges, such as incorporating stochastic extinction and reintroduction of the disease [ref. 19 in the main text]. On the other hand, individual-level models [refs. 20, 21 in the main text] capture the nuanced complexities of human mobility and individual heterogeneity, but are computationally intensive and require granular data for validation.
